# MiR-193b-3p–ERBB4 axis regulates psoriasis pathogenesis via modulating cellular proliferation and inflammatory-mediator production of keratinocytes

**DOI:** 10.1038/s41419-021-04230-5

**Published:** 2021-10-19

**Authors:** Cong Huang, Weilong Zhong, Xuanyao Ren, Xia Huang, Zizhuo Li, Chaofeng Chen, Bin Jiang, Zhenzhen Chen, Xingling Jian, Lili Yang, Xiaoming Liu, Haiyan Huang, Changbing Shen, Xiaofan Chen, Xia Dou, Bo Yu

**Affiliations:** 1 Department of Dermatology, Skin Research Institute of Peking University Shenzhen Hospital, Peking University Shenzhen Hospital, Shenzhen Peking University - The Hong Kong University of Science and Technology Medical Center, Shenzhen, 518036 China; 2 Department of Dermatology, Skin Research Institute of Peking University Shenzhen Hospital, Peking University Shenzhen Hospital, Shenzhen, 518036 China; 3 Biomedical Research Institute, Shenzhen Peking University - The Hong Kong University of Science and Technology Medical Center, Shenzhen, 518036 China

**Keywords:** Mechanisms of disease, miRNAs

## Abstract

Psoriasis is an auto-inflammatory skin disease characterized by abnormal activation of epidermal keratinocytes, aberrant neovascularization, and dysregulation of immune cells. MicroRNAs are small non-coding RNAs that mainly function in the post-transcriptional regulation of gene expression. Recently, accumulating evidence has demonstrated that expression of microRNAs is dysregulated in psoriasis patients and microRNAs play key roles in psoriasis pathogenesis. Downregulation of miR-193b-3p has been identified to be associated with psoriasis development. However, the precise functions and action mechanisms of miR-193b-3p in psoriasis pathogenesis remain unclear. In this study, we confirmed the downregulation of miR-193b-3p in psoriasis patients, psoriasis-like inflammatory cellular models, and an imiquimod (IMQ) -induced mouse model. A negative correlation was found between miR-193b-3p level and patient Psoriasis Area and Severity Index (PASI) score. Furthermore, miR-193b-3p suppressed proliferation, inflammatory-factor secretion, and the STAT3 and NF-κB signaling pathways in keratinocytes. Importantly, intradermal injection of agomiR-193b-3p blocked, whereas antagomiR-193b-3p augmented, the psoriasis-like inflammation in the IMQ-induced mouse model. Bioinformatics analysis and the dual-luciferase reporter assay showed that miR-193b-3p targets *ERBB4* 3ʹ untranslated region (UTR). In addition, *ERBB4* induced proliferation, inflammatory-factor production, and the STAT3 and NF-κB pathways in keratinocytes. Most importantly, forced expression of *ERBB4* could attenuate the effects of miR-193b-3p in keratinocytes, indicating that miR-193b-3p inhibits keratinocyte activation by directly targeting *ERBB4*. In conclusion, our findings demonstrated that the miR-193b-3p–ERBB4 axis underlies the hyperproliferation and aberrant inflammatory-factor secretion of psoriatic keratinocytes, providing a novel, microRNA-related causal mechanism and a potential therapeutic target in psoriasis.

## Introduction

Psoriasis is a chronic and inflammatory dermatosis that affects approximately 3% of the world population, which seriously impairs the life quality of psoriasis patients [1, 2]. Psoriasis is characterized by abnormal proliferation and/or differentiation of keratinocytes, neovascularization, and infiltration of neutrophils, major lymphocytes including T cells, and dendritic cells into epidermis and dermis [1, 2]. Previous reports have shown that internal and external factors, including susceptibility genes and environmental factors contribute to psoriasis pathogenesis [1, 2]. Moreover, it is widely accepted that dysregulation of the crosstalk between epidermal keratinocytes and immune cells plays a critical role in the psoriatic epidermal hyperplasia [3, 4]. Furthermore, several pro-inflammatory factors, such as IL-6, IFN-γ, and TNF-α, have been demonstrated to be central to the interaction between keratinocytes and inflammatory cells, and this interaction might be responsible for the initiation, maintenance, and recurrence of psoriasis [5–8]. However, the precise mechanism underlying psoriasis pathogenesis remains unclear.

MicroRNAs are endogenous, single-stranded, evolutionarily conserved non-coding RNAs (about 22 nucleotides in length), which play key roles in almost all biological processes including proliferation, differentiation, inflammation, and immunity [9–11]. They negatively regulate target genes mainly through mRNA degradation and translational repression by binding to their 3ʹ untranslated regions (UTRs) [9–11]. Previous studies have highlighted the roles of microRNAs in aberrant keratinocyte proliferation and differentiation [12, 13], inflammatory response [14–16], and immune dysfunction [17], all of which contribute to the psoriasis phenotype. Recent studies indicated that the interaction between epidermal keratinocytes and immune cells in psoriasis might be linked via microRNAs/targets axis through regulating chemokine/cytokine production [18, 19]. Such reports mentioned above provide potential insight into the psoriasis pathogenesis and make microRNAs very promising therapeutic targets for psoriasis.

Deep sequencing of microRNAs from human skin tissues revealed that miR-193b-3p is among the top 10/30 downregulated microRNAs in psoriatic skins [20, 21]. In our present study, we confirmed that miR-193b-3p was significantly reduced in psoriatic lesions. Moreover, we showed that decreased miR-193b-3p expression was associated with increased patient Psoriasis Area and Severity Index (PASI) score, suggesting that loss of miR-193b-3p may contribute to psoriasis pathogenesis. Therefore, we further investigated the possible role of miR-193b-3p in psoriasis pathogenesis with the purpose of providing a novel molecular basis and therapeutic target for psoriasis.

Firstly, we performed gain- and loss-of-function assays to explore the role of miR-193b-3p in keratinocytes. We found that miR-193b-3p not only inhibited the proliferation but also reduced the chemokine secretion in keratinocytes. Subsequent experiments revealed that miR-193b-3p inhibited skin inflammation and reduced the disease severity in imiquimod (IMQ)-induced psoriasis mouse model. Mechanism study showed that ERBB4 is highly expressed in psoriasis patient’s epidermis and directly targeted by miR-193b-3p. Through gain-/loss-of-function and gene recovery assays, we proved that ERBB4 is functionally essential and responsible for the biological effects of miR-193b-3p on psoriasis development. Collectively, our findings reveal a link between miR-193b-3p deficiency and ERBB4 protein hyperactivation in psoriasis, which leads to abnormal proliferation and aberrant inflammation of keratinocytes, highlighting the potential use of miR-193b-3p as a novel therapeutic intervention for psoriasis.

## Materials and methods

### Cell lines and cultures

HaCaT cells were obtained from China Center for Type Culture Collection (Wuhan, China) and cultured in DMEM (Gibco, Carlsbad, CA, USA) supplemented with 10% fetal bovine serum (Atlanta Biologicals, Lawrenceville, GA, USA), 100 U/mL penicillin, and 100 µg/mL streptomycin. Normal human keratinocytes (NHKs) were isolated from foreskin as previously described [22]. Firstly, the subcutaneous tissues were removed from the foreskin using sterilized ophthalmic scissors. Then, Dispase II was added to the remaining tissues and incubated at 4 °C overnight. Following the dermis peel off, the epidermis was digested in 0.25% trypsin at 37 °C for 1 h. Finally, the cells were plated in a precoated tissue culture dish. All the cells were incubated at 37 °C in a humidified condition supplemented with 5% CO_2_.

### Reagents, miRNA mimics, siRNAs, and antibodies

TRIzol reagent for RNA extraction was from Invitrogen (Carlsbad, CA, USA). The SYBR Green Master Mix for qPCR was from Bio-Rad (Hercules, CA, USA). MiR-193b-3p mimics/inhibitors, ERBB4 siRNAs, agomiR-193b-3p/antagomiR-193b-3p, as well as their indicated controls were from RiboBio Inc. (Ribobio, Guangzhou, China). The siRNA sequences that targeted ERBB4 were as follows: si-ERBB4#1, 5′-GAAAUCAGCGCAGGAAACAUCUAUA-3′; and si-ERBB4#2, 5′-CCAUCCAGCUGGUUACUCAACUUAU-3′. Antibodies against GAPDH (AF1186), β-actin (AF5003), phospho-NF-κB p65 (Ser311) (AF5878), NF-κB p65 (AF1234), IL-1β (AF7209), and ERBB4 (AF6807) were from Beyotime Biotechnology (China), while phospho-STAT3 (Tyr705) (#9145), phospho-STAT3 (Ser727) (#9134), and STAT3 (#30835) were from Cell Signaling Technology Inc. (Danvers, MA, USA). Peroxidase-conjugated secondary antibody for western blot analysis and immunohistochemistry staining were from Beyotime Biotechnology (A0208). The SuperSignal West Femto Chemiluminescent Substrate Kit used for signaling detection in western blot analysis was from ThermoFisher Scientific (#34095, Rockford, IL, USA). The SignalStain^®^ DAB Substrate Kit for immunohistochemistry signal detection was from Cell Signaling Technology Inc. (#8059, Danvers, MA, USA). All other molecular-grade reagents or chemicals were purchased from Sigma (St Louis, MO, USA) or Fisher (Pittsburgh, PA, USA) unless otherwise mentioned.

### Plasmids/vectors construction

To construct an ERBB4-overexpression vector (ERBB4^OV^), the ERBB4 coding sequence was PCR-amplified from human keratinocyte cDNA using the primer sequences: 5′-ATATCCAGCACAGTGGCGGCCGCATGAAGCCGGCGACAGGA-3′ (Fw) and 5′-GAAGGGCCCTCTAGACTCGAGTTACACCACAGTATTCCGGTGTCT-3′ (Rev). Then the PCR product was sub-cloned into the empty pcDNA4 vector (Invitrogen™ V86320, ThermoFisher). The correct insert was confirmed by DNA sequencing.

The luciferase-based microRNA target expression vectors were constructed using the siCHECK™ Vector system (Promega, USA). Briefly, the cDNA fragment of ERBB4 3′ UTR was amplified and inserted into the firefly luciferase open reading frame of siCHECK™ Vector. Meanwhile, point mutations in the seed region of the predicted miR-193b-3p binding site within the 3′ UTR of ERBB4 were generated and inserted into the siCHECK™ Vector using the ClonExpress II One Step Cloning Kit (C112-01, Vazyme, China). The primers used for ERBB4 3′ UTR amplification were as follows: 5′-AATTCTAGGCGATCGCTCGAGGCTCAGTTGTGGTTTTTTAGGTGG-3′ (Wt-Fw/Mut-Fw1), 5′-CCACTGGGAAGTGTCAAAACTACTGGCCTTGGGG-3′ (Mut-Rev1), 5′-GTTTTGACACTTCCCAGTGGAAGATACAGAGATG-3′ (Mut-Fw2), and 5′-ATTTTATTGCGGCCAGCGGCCGCCCTCTAATGTTCAAGTTAGGTAAGCAC-3′ (Wt-Rev/Mut-Rev2). Wt stands for wild type and Mut for mutant. The correct insert was confirmed by DNA sequencing.

### Patients and specimen collection

All the patients included in the present study (six psoriasis patients and five healthy controls) were recruited from the Department of Dermatology, Peking University Shenzhen Hospital (Shenzhen, China). The psoriasis patients enrolled in this study were not treated with topical therapy for 2 weeks or received systemic treatment for at least 1 month before the skin biopsy. Biopsies (6 mm) were taken from psoriasis patients at the lesional site. Biopsies collected from the non-inflamed skin of healthy subjects were used as controls. The specimens were divided into two parts, one part was immediately placed in liquid nitrogen for RNA extraction, while the other part was fixed with 4% paraformaldehyde and embedded for tissue sectioning. This study was approved by the Ethics Committee of Peking University Shenzhen Hospital, which was conducted in accordance with the Declaration of Helsinki. Informed consents were obtained from all participants.

### Western blot analysis

Western blot was performed as described previously [23, 24]. Briefly, samples were harvested on ice using ice-cold 1× RIPA lysis buffer with premixed protease and phosphatase inhibitor cocktails. Samples (20 µg protein if not specifically indicated) were loaded to a 12% SDS-PAGE gel, fractioned through electrophoresis, and transferred onto PVDF membranes. The membranes were blocked with 5% BSA, and then incubated with appropriate primary and horseradish peroxidase (HRP)-conjugated secondary antibodies. The immunosignal was detected using the SuperSignal West Femto Chemiluminescent Substrate Kit (Thermo Scientific, Rockford, IL, USA). The images were captured using Bio-Rad imaging system and analyzed using Image lab software from Bio-Rad (Hercules, CA, USA).

### Immunohistochemistry staining

ERBB4 (AF6807, Beyotime), STAT3 (#30835, CST), NF-κB (AF1234, Beyotime), S100A8 (AF7929, Beyotime), S100A9 (AF7932, Beyotime), and Ki67 (ab15580, Abcam) levels in the skin tissues were detected using immunohistochemistry as described previously [24]. Briefly, tissues were deparaffinized with xylene, rehydrated with graded ethanol series, and then autoclaved in an unmasking solution (Vector Laboratories, Burlingame, CA, USA) for antigen retrieval before blocking endogenous peroxidase activity with 3% hydrogen peroxide. Tissues were then incubated with blocking buffer at room temperature for 1 h followed by incubation with primary antibodies at 4 °C overnight. Then, biotinylated secondary antibody was added to the tissues and incubated for 30 min at room temperature. Signals were visualized using DAB kit from Cell Signaling Technology Inc. (#8059, Danvers, MA, USA). The sections were counterstained with Mayer’s hematoxylin. The images were captured using Nikon imaging system (Nikon Inc., Melville, NY, USA).

### RNA isolation and quantitative real-time PCR (qRT-PCR)

Total RNA was prepared with TRIzol reagent. RNA concentration was determined using Nanodrop-2000 (Thermo Scientific, Carlsbad, CA, USA). Reverse transcription was done by using high-capacity cDNA reverse transcription kit (Bio-Rad, Hercules, CA, USA). Then the quantitative real-time PCR (qRT-PCR) was used to determine mRNA expression. The final qRT-PCR reaction mix contained 10 μL SYBR Green qPCR Master Mix, 2 μL diluted cDNA, and 8 μL diluted primer mix. The qRT-PCR amplification was performed in a Bio-Rad CFX96 real-time fast PCR system (Hercules, CA, USA). The relative expression of objective gene was calculated using the 2^−ΔΔCt^ method. GAPDH/U6 was used as a loading control. Primer sets used for miR-193b-3p/miR-193a-3p quantification were synthesized by RiboBio Co., Ltd. (Guangzhou, China). All the other primer sets used for qRT-PCR in this study are listed in Table S1.

### Cell transfection, M5 treatment, and cell proliferation assay

Cells were cultured to 70% confluency and then transfected with miR-193b-3p mimics/inhibitors, ERBB4 siRNAs, and indicated controls using Lipofectamine™ RNAiMAX Transfection Reagent (#13778150, Thermo Scientific) according to the manufacturer’s instruction. For the overexpression assay, we used Lipofectamine™ 3000 Transfection Reagent (#L3000015, Thermo Scientific) to deliver ERBB4-overexpression vectors/control vectors into cells. For M5 treatment, cells were added with M5 cocktail, containing recombinant human IL-1α (#200-01 A), IL-17A (#200-17), IL-22 (#200-22), oncostatin M (#300-10), and TNF-α (#300-01 A) (all brought from Peprotech and each at 10 ng/mL), or PBS of equal volume as control and incubated for 24 h. Please note that M5 treatment was used to induce psoriasis-like changes in cultured keratinocytes [25, 26]. Cell proliferation was determined by MTT assay according to the manufacturer’s instruction. Briefly, the MTT solution was added to the cells and incubated for 4 h before DMSO was added to the cultures. A micro plate reader was used to measure the absorbance of dissolved crystals at 490 nm. At least three independent experiments were carried out for each treatment.

### IMQ-induced psoriasis-like mouse model

Female C57BL/6 mice (7 weeks of age) were kept under controlled conditions. The mice were treated with 5% IMQ cream (62.5 mg, Aldara, UK) daily on the shaved and circled back for 5 consecutive days (*n* = 5). Control mice were treated with the same dose of vehicle cream (Vaseline, *n* = 4). All procedures were approved and supervised by Shenzhen Perking University - Hong Kong University of Science and Technology Medical Center Animal Care and Use Committee.

### AgomiR/antagomiR treatment

Female C57BL/6 mice (7 weeks of age, *n* = 8 for each group) were injected intradermally with agomiR-193b-3p/antagomiR-193b-3p (5 nmol in 100 μL PBS), as well as their corresponding controls (agomiR-NC/antagomiR-NC), on day -2 and day -1 before the application of IMQ/Vaseline. AgomiR-193b-3p/antagomiR-193b-3p as well as indicated controls were synthesized by RiboBio Co., Ltd. (Guangzhou, China). The target sequences for agomiR-193b-3p/antagomiR-193b-3p were: sense 5′-AACUGGCCCUCAAAGUCCCGCU-3′ and antisense 5′-AGCGGGACUUUGAGGGCCAGUU-3′. The PASI score of the mouse back skin was recorded daily throughout the whole process of IMQ treatment. The scoring standard of the mouse skin lesion was adjusted according to the clinical PASI score of psoriasis patient: erythema, desquamation, and epidermal thickening were scored 0–4, separately (0 stands for no damage, 1 is considered mild, 2 is moderate, 3 is severe, and 4 is extremely severe), and then added three scores together. That is, the overall scores of the mouse skin lesion range from 0 to 12. For agomiR/antagomiR treatment, all mice were sacrificed on the 6th day and tissues from the skins, spleens, and lymph nodes were collected. All skin tissues were separated into two parts, one part was quickly placed in liquid nitrogen for RNA/protein extraction, while the other part was fixed with 4% paraformaldehyde and embedded for tissue sectioning.

### Bioinformatics analyses

miRDB (http://mirdb.org/miRDB/index.html), TargetScan (http://www.targetscan.org), and PicTar (https://pictar.mdc-berlin.de/cgi-bin/new_PicTar_vertebrate.cgi) were used to predict the target genes of miR-193b-3p.

### Statistics

All experiments were repeated at least three times unless otherwise mentioned. Data are presented as mean ± SEM. Statistical analysis was conducted using GraphPad Prism software (GraphPad Software, Inc. La Jolla, CA, USA). Data were analyzed for significance using one-way ANOVA with Tukey’s post hoc tests. A value of *P* < 0.05 was considered statistically significant.

## Results

### Part 1. miR-193b-3p is downregulated in psoriasis keratinocytes

**Fig. 1:**
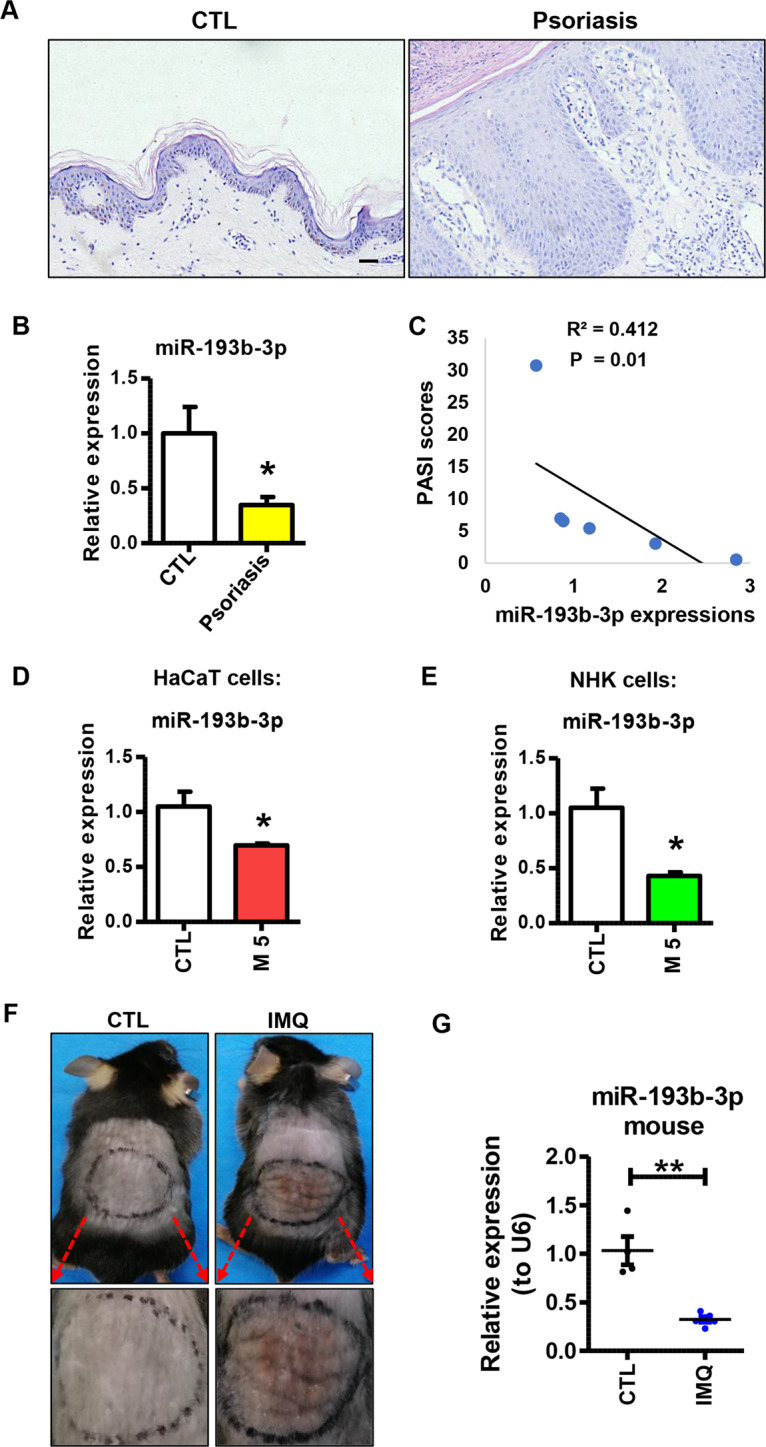
miR-193b-3p is downregulated in psoriasis patients and M5-induced keratinocytes. **A** Representative images showing the pathological morphology of skin tissues derived from psoriasis patients and healthy donors using H&E staining. Scale bar: 50 µm. **B** Expression levels of miR-193b-3p in skin tissues derived from healthy donor (CTL, *n* = 5) and psoriasis patient (Psoriasis, *n* = 6). mRNA levels were analyzed using quantitative real-time PCR (qRT-PCR). U6 was used as the internal control. Each bar represents mean ± SEM. ^*^P < 0.05, compared with the indicated controls. **C** Correlation of miR-193b-3p expression with the PASI score of corresponding patients (*n* = 6). **D** Expression levels of miR-193b-3p in HaCaT cells in the presence or absence of M5 treatment. mRNA levels were analyzed using qRT-PCR. U6 was used as the internal control. Each bar represents mean ± SEM (*n* = 4). ^*^P < 0.05, compared with the indicated controls. **E** Expression levels of miR-193b-3p in normal human keratinocyte cells (NHK cells) in the presence or absence of M5 treatment. mRNA levels were analyzed using qRT-PCR. U6 was used as the internal control. Each bar represents mean ± SEM (*n* = 4). ^*^P < 0.05, compared with the indicated controls. **F** Representative images showing the skin lesions of Vaseline (CTL) and IMQ-treated mice (IMQ). **G** Expression levels of miR-193b-3p in the back skin of Vaseline (CTL) and IMQ-treated mice (IMQ). mRNA levels were analyzed using qRT-PCR. U6 was used as the internal control. Each bar represents mean ± SEM (*n* ≥ 4). ^**^P < 0.01, compared with the indicated controls.

By using deep sequencing, Raaby et al. have reported that miR-193b-3p, a classical tumor suppressor, is downregulated in psoriatic skins [20, 21]. To further confirm this result, we checked miR-193b-3p expression in skin samples derived from psoriasis patients and healthy donors. Compared with the healthy skin, the psoriatic skin showed apparent epidermal hyperplasia and lymphocyte infiltration (Fig. 1A). Additionally, the psoriatic-skin samples showed higher levels of psoriasis-related genes, confirming their psoriatic characteristics at the molecular level (Fig. S1a). Strikingly, miR-193b-3p dramatically decreased in the psoriatic-skin samples (Fig. 1B). Moreover, the miR-193b-3p levels in these samples were negatively correlated with the PASI scores of the corresponding patients (Fig. 1C).

To assess whether miR-193b-3p is dysregulated by psoriasis-related inflammatory factors in vitro, we treated HaCaT cells and NHKs with M5 cocktail to induce psoriasis-like inflammation in these cultured keratinocytes [25, 26], and then measured the expression of miR-193b-3p. As expected, psoriasis-related genes and inflammatory genes were highly expressed in HaCaT cells and NHKs treated with M5 (Fig. S1b, c). Interestingly, miR-193b-3p dramatically decreased in M5-treated HaCaT and NHKs (Fig. 1D, E), indicating that miR-193b-3p expression is attenuated by these inflammatory factors.

IMQ-induced skin inflammation is a well-characterized mouse model for psoriasis [27]. To examine the expression profile of miR-193b-3p in vivo, mice were topically treated with IMQ/Vaseline for 5 consecutive days and sacrificed to harvest skin samples on day 6. As expected, IMQ-treated mice showed increased skin inflammation, while control (CTL) mice did not show any signs of skin lesion (Fig. 1F). Unexpectedly, IMQ significantly decreased miR-193b-3p in mouse skin (Fig. 1G), demonstrating that miR-193b-3p is inhibited in a psoriasis-like inflammatory environment. Together, these results verify that miR-193b-3p is downregulated in psoriatic keratinocytes, suggesting a potential role of miR-193b-3p in psoriasis pathogenesis.

### Part 2. miR-193b-3p inhibits keratinocyte activation in vitro

**Fig. 2:**
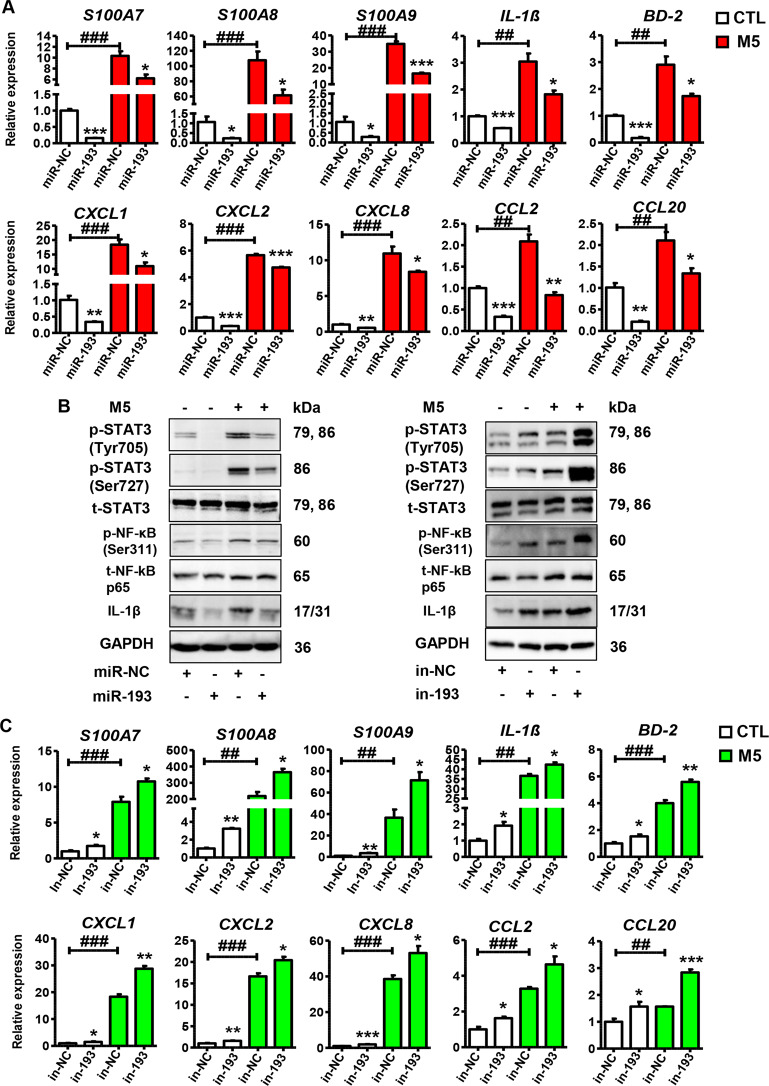
Function of miR-193b-3p in M5-induced HaCaT cells. **A** Expression levels of psoriasis-related genes and inflammatory genes in HaCaT cells transfected with miR-193b-3p mimics (miR-193)/control (miR-NC) in the presence or absence of M5 treatment. mRNA levels were analyzed using qRT-PCR. GAPDH was used as the internal control. Each bar represents mean ± SEM (*n* = 3). ^***^P < 0.001, ^**^P < 0.01, ^*^P < 0.05, ^###^*P* < 0.001, ^##^*P* *<* 0.01, compared with the indicated controls. **B** Representative blots showing the levels of phospho-STAT3 (Tyr705), phospho-STAT3 (Ser727), phospho-NF-κB p65 (Ser311), IL-1β, etc. in HaCaT cells transfected with miR-193b-3p mimics (miR-193) or miR-193b-3p inhibitors (in-193) and their indicated CTLs (miR-NC/in-NC) in the presence or absence of M5 treatment. Protein levels were detected by western blotting. GAPDH was used as a protein loading control. **C** Expression levels of psoriasis-related genes and inflammatory genes in HaCaT cells transfected with miR-193b-3p inhibitors (in-193)/control (in-NC) in the presence or absence of M5 treatment. mRNA levels were analyzed using qRT-PCR. GAPDH was used as the internal control. Each bar represents mean ± SEM (*n* = 3). ^***^P < 0.001, ^**^P < 0.01, ^*^P < 0.05, ^###^*P* < 0.001, ^##^*P* < 0.01, compared with the indicated controls.

To explore the possible role of miR-193b-3p in psoriasis pathogenesis, we used miR-193b-3p mimics/inhibitors to overexpress/inhibit miR-193b-3p in HaCaT cells. As expected, miR-193b-3p mimics dramatically increased, while miR-193b-3p inhibitors dramatically decreased, miR-193b-3p expression in HaCaT cells (Fig. S2a). Meanwhile, miR-193b-3p mimics reduced, while miR-193b-3p inhibitors promoted, cellular proliferation (Fig. S2b). We then introduced M5 into our system to induce a psoriasis-like phenotype in HaCaT cells. As shown in Fig. 2A, psoriasis-related genes in HaCaT cells, such as *S100A7*, *S100A8*, and *S100A9*, were downregulated by miR-193b-3p overexpression in the presence or absence of M5 treatment. Unexpectedly, pro-inflammatory genes, including *CXCL1*, *CCL2*, and *IL-1β*, which may lead to severe inflammation in psoriasis patients, were downregulated by miR-193b-3p mimics in the presence or absence of M5 treatment (Fig. 2A). Moreover, miR-193b-3p overexpression dramatically decreased the levels of phospho-STAT3 (Tyr705), phospho-STAT3 (Ser727), and phospho-NF-κB p65 (Ser311) in HaCaT cells (Fig. 2B), suggesting that miR-193b-3p not only decreases inflammatory-factor secretion but also inhibits the inflammatory STAT3 and NF-κB pathways. To confirm the regulatory role of miR-193b-3p in keratinocytes, miR-193b-3p inhibitors were used to block the endogenous miR-193b-3p expression in HaCaT cells. As shown in Fig. 2B, C, miR-193b-3p inhibitors significantly promoted the expression of psoriasis-related genes and inflammatory genes, as well as the STAT3 and NF-κB pathways in the presence or absence of M5 treatment. By gain- and loss-of-function assays, we observed similar results regarding the role of miR-193b-3p in NHKs (Fig. S3). All these data indicate that miR-193b-3p inhibits the keratinocyte activities in vitro via suppressing its proliferation, inflammatory-factor production, and inflammatory pathways.

### Part 3. Role of miR-193b-3p in the IMQ-induced psoriasis mouse model

**Fig. 3:**
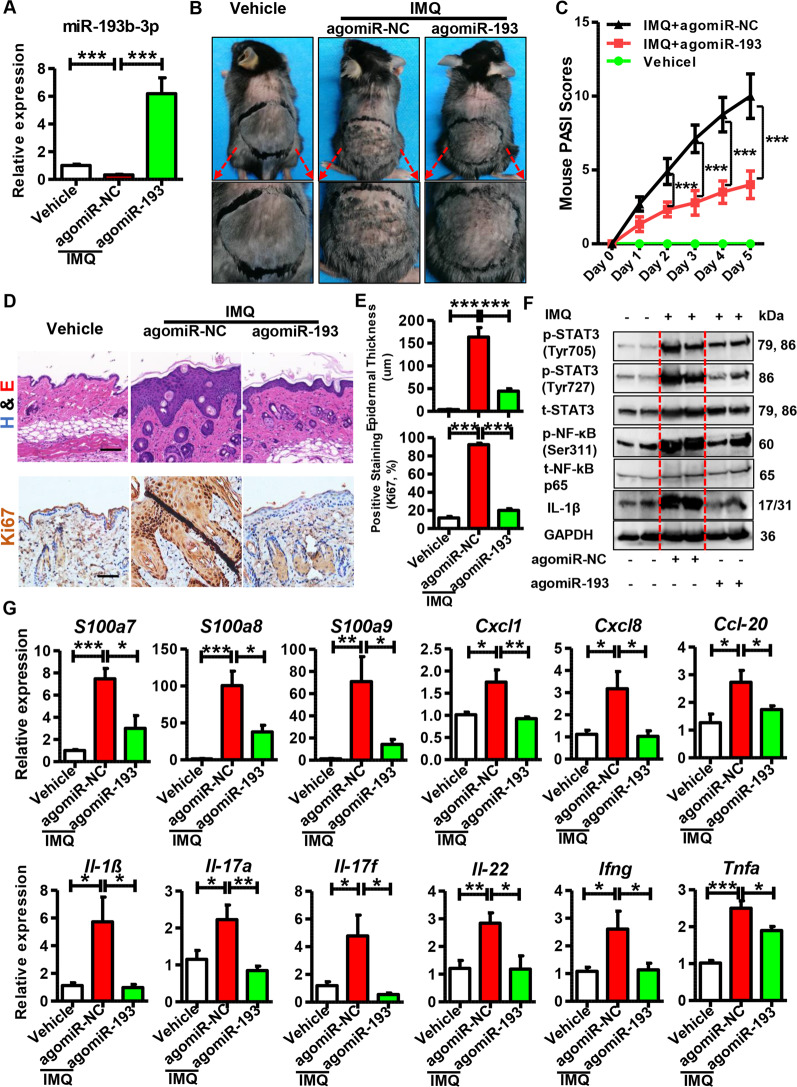
Function of agomiR-193b-3p in IMQ-induced psoriasis mouse model. **A** Expression levels of miR-193b-3p in the mouse back skin of different groups. Vehicle means mice injected with agomiR-NC and treated with Vaseline; agomiR-NC + IMQ and agomiR-193 + IMQ means mice injected with agomiR-NC and agomiR-193b-3p, respectively,and then treated with IMQ for 5 consecutive days. histologicalmiR-193b-3p levels were analyzed using qRT-PCR. U6 was used as the internal control. Each bar represents mean ± SEM (*n* = 8). ^***^P < 0.001, compared with the indicated controls. **B** Representative pictures of the mouse back skins for agomiR-193b-3p injection or agomiR-NC injection with or without IMQ treatment for 5 consecutive days. **C** PASI scores of mouse back skin lesions in different groups at different time points. PASI scores of mouse back skin lesions were recorded and analyzed daily. Each bar represents mean ± SEM (*n* = 8). ^***^P < 0.001, compared with the indicated controls. **D** Representative images showing histological feature of skin tissues derived from mouse back injected with agomiR-193 or agomiR-NC in the presence or absence of IMQ treatment for 5 consecutive days. Scar bar of upper panel: 150 µm, scar bar of lower panel: 100 µm. **E** Quantification data of skin epidermal thickness (upper panel, *n* = 8) and Ki67-positive cells (lower panel, *n* = 8) in skin tissues derived from mouse back injected with agomiR-193 or agomiR-NC in the presence or absence of IMQ treatment for 5 consecutive days. ^***^P < 0.001, compared with the indicated controls. **F** Representative blots showing the levels of phospho-STAT3 (Tyr705), phospho-STAT3 (Ser727), phospho-NF-κB p65 (Ser311), IL-1β, etc. in skin tissues derived from mouse back injected with agomiR-193 or agomiR-NC in the presence or absence of IMQ treatment for 5 consecutive days. Tissues were collected at day 6 and protein levels were detected by western blotting. GAPDH was used as a protein loading control. **G** mRNA levels of psoriasis-related genes and inflammatory genes in skin tissues derived from mouse back injected with agomiR-193 or agomiR-NC in the presence or absence of IMQ treatment for 5 consecutive days. Skin tissues were collected at day 6 and mRNA levels were analyzed using qRT-PCR. GAPDH was used as the internal control. Each bar represents mean ± SEM (*n* = 8 for each group). ^***^P < 0.001, ^**^P < 0.01, ^*^P < 0.05 compared with the indicated controls.

To examine the role of miR-193b-3p in vivo, we intradermally injected agomiR-193b-3p (synthetic miR-193b-3p) into the shaved back of mice. Then, mice were topically treated with IMQ/Vaseline as mentioned in section “Materials and methods” (Fig. S4a). As expected, miR-193b-3p was significantly downregulated in agomiR-NC + IMQ group, compared with the vehicle group (agomiR-NC + Vaseline), while the intradermal injection of agomiR-193b-3p dramatically increased miR-193b-3p expression (Fig. 3A). Meanwhile, the IMQ-treated mice showed increased skin lesions and higher PASI scores than the vehicle group, whereas agomiR-193b-3p significantly inhibited IMQ-induced skin inflammation, as indicated by improved skin lesions (Fig. 3B) and reduced PASI scores (Fig. 3C). Accordingly, histological analyses and Ki67 staining showed that IMQ induced thickened and hyperplastic epidermis, whereas agomiR-193b-3p significantly decreased the IMQ-induced excessive proliferation of keratinocytes (Fig. 3D, E). Additionally, IMQ treatment increased, whereas agomiR-193b-3p significantly decreased, the levels of phospho-STAT3 (Tyr705), phospho-STAT3 (Ser727), and phospho-NF-κB p65 (Ser311) in mouse skins (Fig. 3F). Furthermore, immunohistochemistry analyses showed that IMQ increased, whereas agomiR-193b-3p significantly decreased, the STAT3 and NF-κB levels in the skins (Fig. S4c). In addition, agomiR-193b-3p decreased the expression of psoriasis-related genes and inflammatory genes (Figs. 3G and S4b). Moreover, agomiR-193b-3p significantly downregulated the expression of Th1 (*Ifng*, *Tnfα*), Th17 (*Il17a*, *Il17f*), and Th22 (*Il22*) cell markers in mouse skin (Fig. 3G), suggesting suppressed lymphocyte infiltration. Interestingly, agomiR-193b-3p decreased both the spleen size and the splenic Il-1β expression (Fig. S4d,e,f), indicating that agomiR-193b-3p helps torelieve the systemic inflammatory response in IMQ-treated mice. Notably, decreased Il17 and Il22 were observed in the lymph nodes of the agomiR-193b-3p + IMQ group (Fig. S4g), demonstrating that agomiR-193b-3p systemically reduced the immune response in the IMQ-induced mice. The above results demonstrate that miR-193b-3p overexpression inhibits psoriasis development in vivo.

**Fig. 4:**
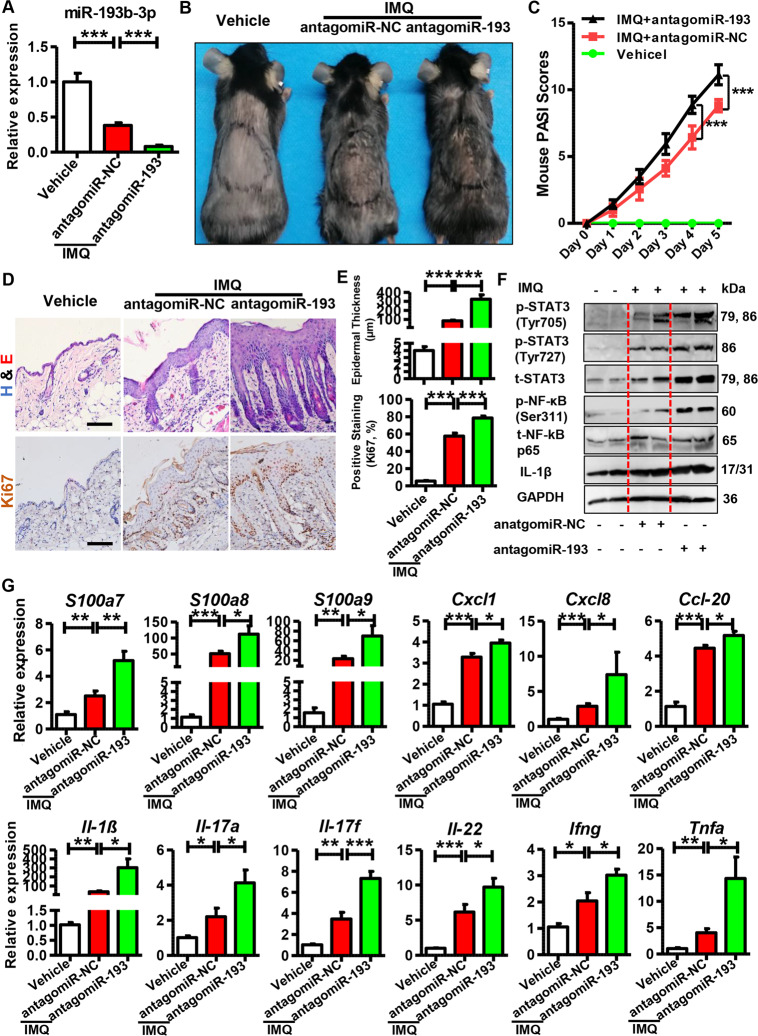
Role of antagomiR-193b-3p in IMQ-induced psoriasis mouse model. **A** Expression levels of miR-193b-3p in mouse back skin of different groups. Vehicle means mice injected with antagomiR-NC and treated with Vaseline, antagomiR-NC + IMQ and antagomiR-193 + IMQ means mice injected with antagomiR-NC and antagomiR-193b-3p, respectively, and then treated with IMQ for 5 consecutive days. miR-193b-3p levels were analyzed using qRT-PCR. U6 was used as the internal control. Each bar represents mean ± SEM (*n* = 8). ^***^P < 0.001, compared with the indicated controls. **B** Representative pictures showing the mouse back skin lesions injectied withantagomiR-193/antagomiR-NC in the presence or obsence of IMQ treatment for 5 consecutive days. **C** PASI scores of mouse back skin lesions in different groups at different time points. PASI scores of mouse back skin lesions were recorded and analyzed daily. Each bar represents mean ± SEM (*n* = 8). ^***^P < 0.001, compared with the indicated controls. **D** Representative images showing histological feature of skin tissues derived from mouse back injected with antagomiR-193 or feature of skin tissues derived from mouse back injected with antagomiR-193 or antagomiR-NC in the presence or absence of IMQ treatment for 5 consecutive days. Scar bar of upper panel: 100 µm, scar bar of lower panel: 100 µm. **E** Quantification data of skin epidermal thickness (upper panel, *n* = 8) and Ki67-positive cells (lower panel, *n* = 8) in skin tissues derived from mouse back injected with antagomiR-193 or antagomiR-NC in the presence or absence of IMQ treatment for 5 consecutive days. ^***^P < 0.001, compared with the indicated controls. **F** Representative blots showing the levels of phospho-STAT3 (Tyr705), phospho-STAT3 (Ser727), phospho-NF-κB p65 (Ser311), IL-1β, etc. in skin tissues derived from mouse back injected with antagomiR-193 or antagomiR-NC in the presence or absence of IMQ treatment for 5 consecutive days. Tissues were collected at day 6 and protein levels were detected by western blotting. GAPDH was used as a protein loading control. **G** mRNA levels of psoriasis-related genes and inflammatory genes in skin tissues derived from mouse back injected with antagomiR-193 or antagomiR-NC in the presence or absence of IMQ treatment for 5 consecutive days. Skin tissues were collected at day 6 and mRNA levels were analyzed using qRT-PCR. GAPDH was used as the internal control. Each bar represents mean ± SEM (*n* = 8 for each group). ^***^P < 0.001, ^**^P < 0.01, ^*^P < 0.05 compared with the indicated controls.

To confirm the effects of miR-193b-3p in psoriasis development, we intradermally injected antagomiR-193b-3p (synthetic molecules that block endogenous miR-193b-3p expression in vivo) into the shaved back of mice before IMQ treatment. As shown in Fig. 4A, miR-193b-3p was dramatically reduced in the antagomiR-193b-3p + IMQ group, compared with the antagomiR-NC + IMQ group. Meanwhile, there was a significant increase in skin inflammation and severity score on day 5 following antagomiR-193b-3p treatment (Fig. 4B, C). Additionally, IMQ induced epidermal thickening and hyperplasia, which was further enhanced by antagomiR-193b-3p (Fig. 4D, E). Moreover, antagomiR-193b-3p upregulated the levels of phospho-STAT3 (Tyr705), phospho-STAT3 (Ser727), and phospho-NF-κB p65 (Ser311) in the IMQ-induced mouse skin (Fig. 4F), which is further confirmed by the immunohistochemistry results (Fig. S5a, b). Furthermore, mice injected with antagomiR-193b-3p expressed higher levels of psoriasis-related genes (Fig. 4G). Notably, the qRT-PCR analyses for the Th1, Th22, and Th17 cell markers showed that antagomiR-193b-3p markedly increased the cutaneous inflammatory cell infiltration (Fig. 4G). In accordance with this result, the spleen size and splenic *Il-1β* expression in the antagomiR-193b-3p administration group were dramatically increased, compared with the antagomiR-NC + IMQ/Vehicle group (Fig. S5c,d,e). In addition, antagomiR-193b-3p significantly upregulated *Il17* and *Il22* in lymph nodes (Fig. S5f). Together, these results indicated that miR-193b-3p inhibition in vivo promotes immunopathological changes in psoriasis and accelerates disease severity.

### Part 4. miR-193b-3p inhibits the keratinocyte activities by targeting ERBB4

**Fig. 5:**
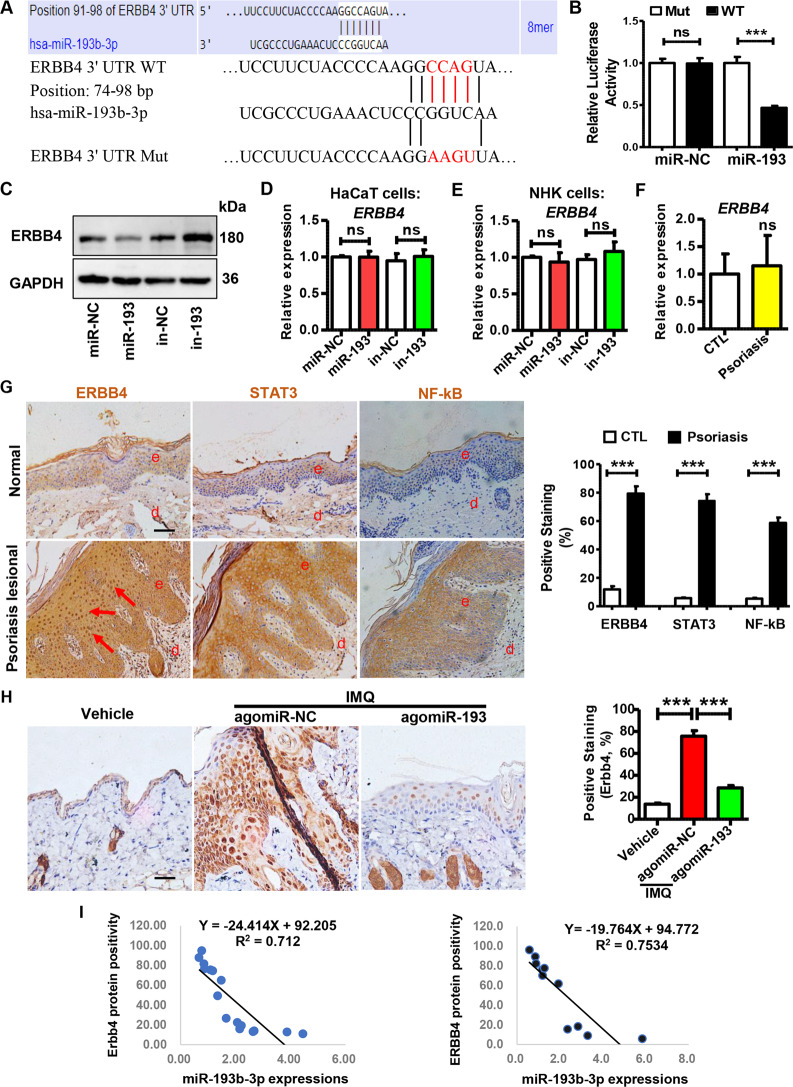
miR-193b-3p directly targets ERBB4. **A** Sequence alignment of wild-type (WT) and mutated (Mut) putative miR-193b-3p-binding sites in the 3ʹ UTR of ERBB4. **B** Relative luciferase activities of plasmids carrying WT or mutant ERBB4 3ʹ UTR in 293 T cells co-transfected with miR-193b-3p mimics or miR-NC. Each bar represents mean ± SEM (*n* = 6). ^***^P < 0.001, ns: no significant difference, compared with the indicated controls. **C** Representative blots showing protein levels of ERBB4 in HaCaT cells transfected with miR-193b-3p mimics (miR-193) or miR-193b-3p inhibitors (in-193) and their indicated CTLs (miR-NC and in-NC). Protein levels were detected by western blotting. GAPDH was used as a protein loading control. **D** The mRNA levels of *ERBB4* in HaCaT cells transfected with miR-193b-3p mimics (miR-193) or miR-193b-3p inhibitors (in-193) were examined by qRT-PCR. Each bar represents mean ± SEM (*n* = 3). ns: no significant difference, compared with the indicated controls. **E** The mRNA levels of *ERBB4* in NHKs transfected with miR-193b-3p mimics (miR-193) or miR-193b-3p inhibitors (in-193) were examined by qRT-PCR. Each bar represents mean ± SEM (*n* = 3). ns: no significant difference, compared with the indicated controls. **F** The mRNA levels of *ERBB4* in skin tissues derived from healthy donors (CTL, *n* = 5) and psoriasis patients (Psoriasis, *n* = 6) were examined by qRT-PCR. GAPDH was used as the internal control. Each bar represents mean ± SEM. ns: no significant difference, compared with the indicated controls. **G** Left panel: ERBB4, STAT3, and NF-κB levels were analyzed in healthy (Normal) and psoriasis lesional skin samples (Psoriasis lesional) using immunohistochemistry. Scale bar = 50 µm. Right panel: quantification of ERBB4, STAT3, and NF-κB positivity in clinical samples (Normal control, *n* = 5; Psoriasis lesional, *n* = 6). Each bar represents mean ± SEM. ^***^P < 0.001, compared with the indicated controls. **H** Left panel: Erbb4 levels were analyzed in mouse back skins of different groups using immunohistochemistry. Scale bar = 50 µm. Right panel: quantification of Erbb4 positivity in the skin tissues derived from different groups. Each bar represents mean ± SEM (*n* = 8 for each group). ^***^P < 0.001, compared with the indicated controls. Vehicle means mice injected with agomiR-NC and treated with Vaseline, agomiR-NC + IMQ and agomiR-193 + IMQ means mice injected with agomiR-NC and agomiR-193, respectively, and then treated with IMQ for 5 consecutive days. **I** Left panel: correlation of miR-193b-3p expression and Erbb4 protein positivity in IMQ-induced psoriasis mouse model (*n* = 16). Right panel: correlation of miR-193b-3p expression and ERBB4 protein positivity in clinical samples (*n* = 11).

To gain insight into the molecular mechanism by which miR-193b-3p regulates psoriasis pathogenesis, we combined three bioinformatics tools to predict the putative target of miR-193b-3p. As shown in Figs. 5A and S6a,b,c*ERBB4*, which has been reported to be involved in tumorigenesis and positively correlated with keratinocyte proliferation and epidermal thickness [28–30], was predicted to be a miR-193b-3p target. To validate this target gene, we generated a luciferase reporter construct that contained the wild-type (WT) 3ʹ UTR of the *ERBB4* gene, and a mutant (Mut) reporter construct in which the binding site of miR-193b-3p was mutated (Fig. 5A). Compared with the miR-NC-transfected cells, miR-193b-3p mimics significantly inhibited luciferase activity of cells co-transfected with the *ERBB4* WT 3ʹ UTR construct, but this inhibition was lost when co-transfected with the Mut construct (Fig. 5B). We next sought to evaluate whether miR-193b-3p influences endogenous *ERBB4* expression in keratinocytes. Notably, ERBB4 protein levels were decreased by miR-193b-3p mimics, whereas increased by miR-193b-3p inhibitors in keratinocytes (Fig. 5C). Unexpectedly, neither miR-193b-3p mimics nor miR-193b-3p inhibitors affected *ERBB4* mRNA levels in HaCaT cells or NHKs (Fig. 5D, E). Moreover, the *ERBB4* mRNA levels in the skin tissues derived from the healthy donors were not significantly different from those derived from psoriasis patients (Fig. 5F). Strikingly, the protein levels of ERBB4, as well as STAT3 and NF-κB, were significantly upregulated in lesional skin samples from psoriasis patients (Fig. 5G). In addition, the Erbb4 protein in the skins from the agomiR-193b-3p + IMQ group was dramatically decreased, compared with that from the agomiR-NC + IMQ group (Figs. 5H and S6d). However, the *Erbb4* mRNA was not affected by agomiR-193b-3p (Fig. S6e), suggesting that miR-193b-3p regulates *Erbb4* expression post-transcriptionally. More importantly, negative correlations of miR-193b-3p expression and ERBB4 protein positivity were observed both in the IMQ-induced mouse model and clinical samples (Fig. 5I). Together, data from luciferase reporter assay, transfection experiments, immunohistochemistry analysis, and clinical samples indicate that ERBB4 is a direct target of miR-193b-3p.

### Part 5. ERBB4 plays an important role in keratinocytes

**Fig. 6:**
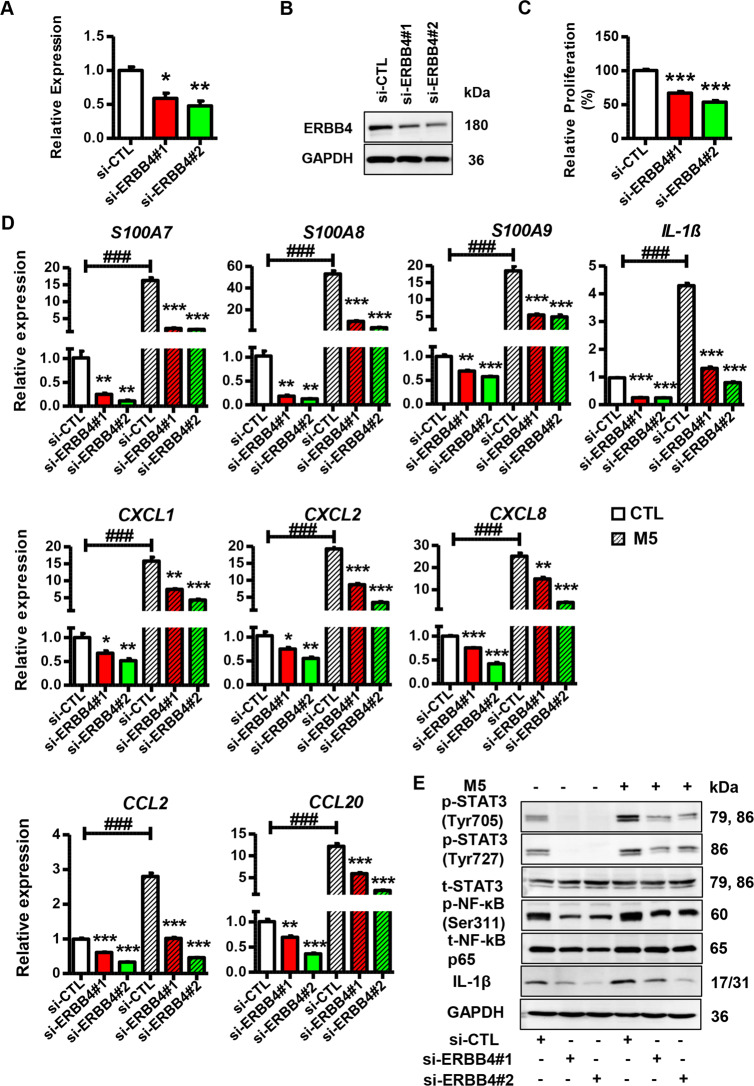
Function of ERBB4 knock down in keratinocytes. **A** Expression levels of ERBB4 in HaCaT cells transfected with ERBB4 siRNAs or si-CTL. si-ERBB4#1 and si-ERBB4#2 means two siRNAs specifically targeting ERBB4, while si-CTL means control for interference RNAs. Cells were collected 2 days after transfection and mRNA levels were analyzed using qRT-PCR. GAPDH was used as the internal control. Each bar represents mean ± SEM (*n* = 3). ^**^P < 0.01, ^*^P < 0.05, compared with the indicated controls. **B** Representative blots showing protein levels of ERBB4 in HaCaT cells transfected with ERBB4 siRNAs or si-CTL. Protein levels were detected by western blotting. GAPDH was used as a protein loading control. **C** Relative proliferation rate of HaCaT cells transfected with ERBB4 siRNAs or si-CTL. Cell proliferation was analyzed using MTT assay. Each bar represents mean ± SEM (*n* = 3). ^***^P < 0.001, compared with the indicated controls. **D** The mRNA levels of psoriasis-related genes and inflammatory genes in HaCaT cells transfected with ERBB4 siRNAs or si-CTL in the presence or absence of M5 treatment. Cells were collected 2 days after transfection and mRNA levels were analyzed using qRT-PCR. GAPDH was used as the internal control. Each bar represents mean ± SEM (*n* = 3). ^***^P < 0.001, ^**^P < 0.01, ^*^P < 0.05, ^###^*P* < 0.001 compared with the indicated controls. **E** Representative blots showing the levels of phospho-STAT3 (Tyr705), phospho-STAT3 (Ser727), phospho-NF-κB p65 (Ser311), and IL-1β in HaCaT cells transfected with ERBB4 siRNAs or si-CTL in the presence or absence of M5 treatment. Protein levels were detected by western blotting. GAPDH was used as a protein loading control.

**Fig. 7:**
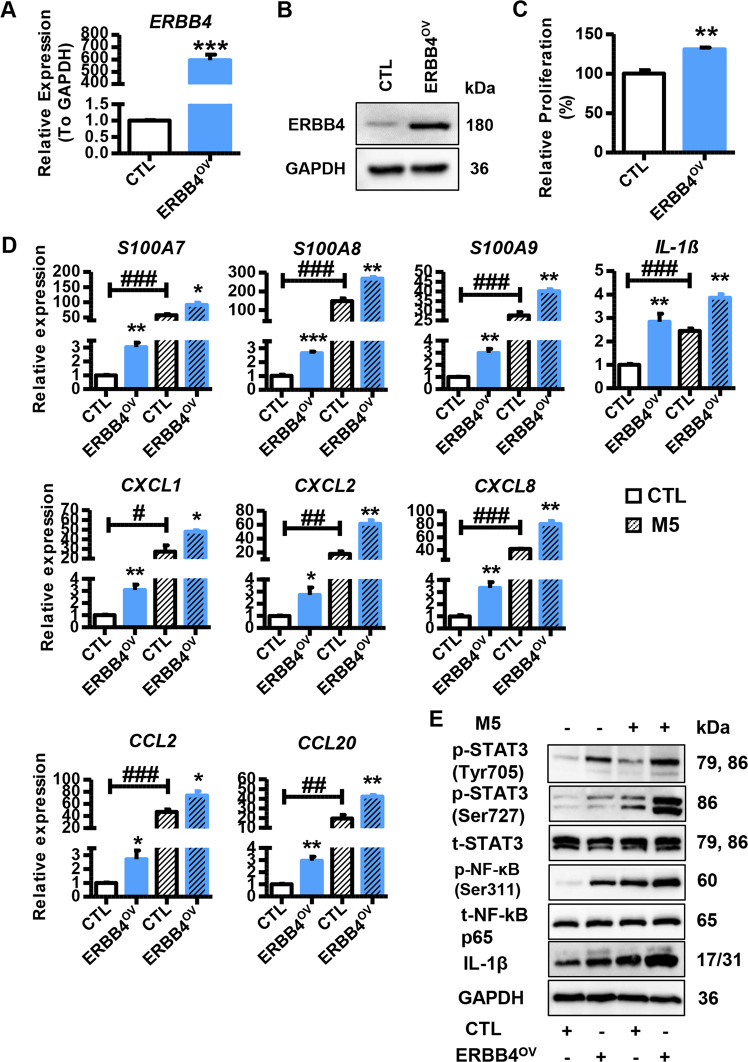
Function of ERBB4 overexpression in keratinocytes. **A** Expression levels of *ERBB4* in HaCaT cells transfected with ERBB4-overexpression vector (ERBB4^OV^) or indicated control (CTL). Cells were collected 2 days after transfection and mRNA levels were analyzed using qRT-PCR. GAPDH was used as the internal control. Each bar represents mean ± SEM (*n* = 3). ^***^P < 0.001, compared with the indicated controls. **B** Representative blots showing protein levels of ERBB4 in HaCaT cells transfected with ERBB4^OV^ or CTL. Protein levels were detected by western blotting. GAPDH was used as a protein loading control. **C** Relative proliferation rate of HaCaT cells transfected with ERBB4^OV^ or CTL. Cell proliferation was analyzed using MTT assay. Each bar represents mean ± SEM (*n* = 3). ^**^P < 0.01, compared with the indicated controls. **D** The mRNA levels of psoriasis-related genes and inflammatory genes in HaCaT cells transfected with ERBB4^OV^ or CTL in the presence or absence of M5 treatment. Cells were harvested 2 days after transfection and mRNA levels were analyzed using qRT-PCR. GAPDH was used as the internal control. Each bar represents mean ± SEM (*n* = 3). ^**^P < 0.01, ^*^P < 0.05, ^###^*P* < 0.001, ^##^*P* < 0.01, ^#^*P* < 0.05, compared with the indicated controls. **E** Representative blots showing the levels of phospho-STAT3 (Tyr705), phospho-STAT3 (Ser727), phospho-NF-κB p65 (Ser311), and IL-1β in the HaCaT cells transfected with ERBB4^OV^ or CTL in the presence or absence of M5 treatment. Protein levels were detected by western blotting. GAPDH was used as a protein loading control.

To explore the biological role and importance of ERBB4 in psoriasis development, we performed a loss-of-function assay by transfecting keratinocytes with ERBB4 siRNAs. As shown in Fig. 6A, B, specific siRNAs dramatically decreased ERBB4 expression in keratinocytes. Additionally, we detected reduced proliferation in the keratinocytes with ERBB4 knockdown (Fig. 6C). Strikingly, ERBB4 knockdown inhibited the expression of psoriasis-related genes and inflammatory genes in the presence or absence of M5 treatment (Fig. 6D). Moreover, si-ERBB4 decreased the levels of phospho-STAT3 (Tyr705), phospho-STAT3 (Ser727), and phospho-NF-κB p65 (Ser311) in keratinocytes in the presence or absence of M5 treatment (Fig. 6E). Then, we performed a gain-of-function assay by transfecting keratinocytes with ERBB4-overexpression vector. As expected, the ERBB4-overexpression vector dramatically upregulated ERBB4 in keratinocytes (Fig. 7A, B). Additionally, ERBB4 overexpression promoted keratinocyte proliferation (Fig. 7C). Furthermore, ERBB4 overexpression increased the expression of psoriasis-related genes and inflammatory genes, and the levels of phospho-STAT3 (Tyr705), phospho-STAT3 (Ser727), and phospho-NF-κB p65 (Ser311) in the presence or absence of M5 treatment (Fig. 7D, E). From the aforementioned results, it could be concluded that ERBB4 acts as a potential mediator of the effects of miR-193b-3p on the proliferation and inflammation in keratinocytes.

**Fig. 8:**
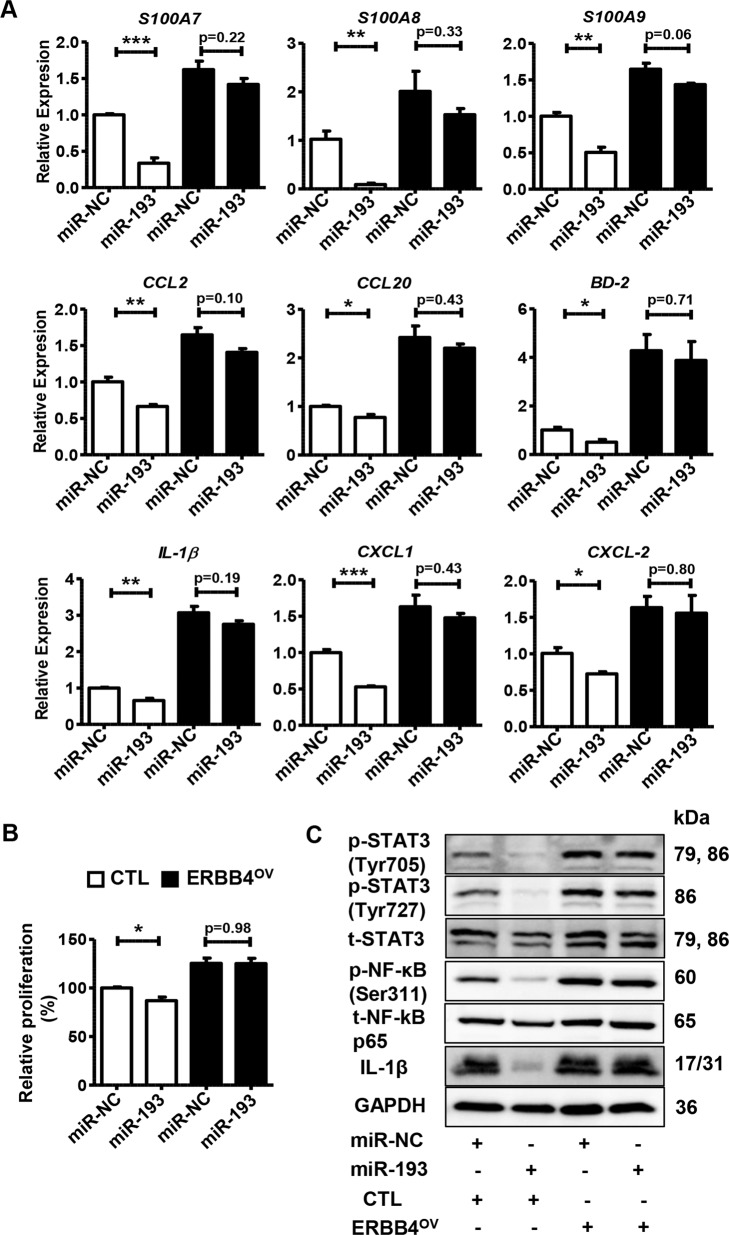
ERBB4 rescues the inhibitory effects of miR-193b-3p on keratinocytes. **A** The mRNA levels of psoriasis-related genes and inflammatory genes in HaCaT cells co-transfected with miR-193b-3p mimics and ERBB4-overexpression vector (ERBB4^OV^). Cells were collected 2 days after co-transfection and mRNA levels were analyzed using qRT-PCR. GAPDH was used as the internal control. Each bar represents mean ± SEM (*n* = 3). ^***^P < 0.001, ^**^P < 0.01, ^*^P < 0.05, compared with the indicated controls. **B** Relative proliferation rate of HaCaT cells co-transfected with miR-193/miR-NC and ERBB4^OV^/CTL. Cell proliferation was analyzed using MTT assay. Each bar represents mean ± SEM (*n* = 3). ^*^P < 0.05, compared with the indicated controls. **C** Representative blots showing the levels of phospho-STAT3 (Tyr705), phospho-STAT3 (Ser727), phospho-NF-κB p65 (Ser311), and IL-1β in the keratinocytes co-transfected with miR-193/miR-NC and ERBB4^OV^/CTL. Protein levels were detected by western blotting. GAPDH was used as a protein loading control.

To further investigate the relationship between miR-193b-3p and ERBB4, we performed gene recovery experiments. Briefly, we co-transfected miR-193b-3p mimics (or miR-NC) with ERBB4-overexpression vector (or control) into keratinocytes and then performed qRT-PCR and western blot analysis. Interestingly, ERBB4 overexpression could abolish the inhibitory effects of miR-193b-3p on cellular proliferation and inflammation (Fig. 8A, B). Moreover, miR-193b-3p mimics could no longer render the inflammatory signals in keratinocytes with ERBB4 overexpression (Fig. 8C). From a sideways approach, our data demonstrated that ERBB4 overexpression neutralized the effects of miR-193b-3p, further indicating that miR-193b-3p regulates keratinocyte activation by directly targeting ERBB4.

## Discussion

Psoriasis is thought to result from impaired communication between keratinocytes and immune cells, because of genetic and environmental factors [8]. MicroRNAs are short non-coding RNAs that mainly regulate gene expression post-transcriptionally [9–11]. Recently, dysregulated microRNAs have been uncovered in psoriasis, indicating the central role of these microRNAs and their potential as diagnostic biomarkers in psoriasis [31, 32]. Currently, most researchers focus on the study of microRNAs in the abnormal proliferation and/or differentiation of keratinocytes, and dysregulation of immune cells, including T lymphocytes and dendritic cells, whereas relatively less attention is paid to the effects of microRNAs on the interaction between keratinocytes and immune cells. However, an in-depth understanding of the regulatory roles of microRNAs in psoriasis inflammation is essential for the development of new therapeutics for treating this auto-inflammatory disease.

By using next-generation sequencing, Raaby et al. have found that miR-193b-3p is downregulated in the skin lesions, especially in the epidermal keratinocytes, of psoriasis patients [20, 21]. In our in vitro system, M5 treatment, which was used to mimic the function of inflammatory mediators to keratinocytes [25, 26], dramatically reduced the miR-193b-3p expression (Fig. 1D, E). Moreover, in IMQ-induced mouse skin, IMQ treatment significantly decreased miR-193b-3p expression (Fig. 1F, G). Therefore, inflammatory factors inhibit miR-193b-3p expression, but the mechanism by which inflammatory mediators regulate miR-193b-3p expression is unclear. Yan et al. have reported that activated NF-κB, which is commonly observed in psoriasis patients and the psoriasis-like mouse model, induces miR-31 transcription in keratinocytes [33], suggesting that inflammation/inflammatory pathways may affect the transcription of microRNA genes. However, the specific molecular mechanism whereby inflammatory cytokines suppress miR-193b-3p expression still needs to be elucidated.

In psoriatic skins, infiltrated immune cells produce inflammatory mediators that lead to the activation of keratinocytes, resulting in epidermal hyperplasia [1, 8, 34]. As the “initiator” of psoriasis, epidermal keratinocytes, especially activated keratinocytes, secrete large numbers of inflammatory factors that function as important signal transmitters between keratinocytes and immune cells. Therefore, disruption of the crosstalk between keratinocytes and the immune system by modulating the inflammatory cytokines/chemokines in keratinocytes is expected to be a novel target for new drug development against psoriasis. In addition to the tumor suppressor role, miR-193b-3p also plays a vital role in the inflammatory process. For instance, in mouse chondrocytes, miR-193b-3p downregulates TNF-α [35], which is an important inflammatory factor in psoriasis pathogenesis, suggesting that dysregulated miR-193b-3p in psoriatic lesions may also contribute to the psoriatic inflammatory response. To explore the role of miR-193b-3p in psoriasis pathogenesis, we performed gain-/loss-of-function assays in keratinocytes. Strikingly, miR-193b-3p overexpression significantly inhibited, whereas miR-193b-3p inhibition significantly promoted, the expression of inflammatory genes in keratinocytes. Additionally, miR-193b-3p dramatically decreased the levels of phospho-STAT3 (Tyr705), phospho-STAT3 (Ser727), and phospho-NF-κB p65 (Ser311) (Figs. 2 and S3), indicating that miR-193b-3p not only reduces the production of inflammatory factors but also blocks the STAT3 and NF-κB pathways, which are two important pathways that affect the inflammatory response in psoriasis pathogenesis [36, 37]. Interestingly, there is evidence showing that miR-193a-3p, which is a sister microRNA of miR-193b-3p and shares similar mature sequences as that of miR-193b-3p (Table S2), also plays crucial roles in regulating the cellular inflammation through downregulation of inflammatory factors, including IL-1β, TNF-α, and NF-κB [38, 39]. In fact, our parallel experimental results confirmed that miR-193a-3p has functions similar to that of miR-193b-3p in HaCaT cells (Fig. S7). However, we did not detect any significant changes in the expression of miR-193a-3p in our in vitro or in vivo models (Fig. S8). The genes encoding these two microRNAs are located on two different chromosomes (Table S2), indicating that their different expression profiles can be attributed to their different transcription mechanisms.

In the IMQ-induced mouse model, the activities of epidermal keratinocytes were inhibited by agomiR-193b-3p, as indicated by reduced expression of psoriasis-related genes and decreased inflammatory-mediator production (Fig. 3). Moreover, miR-193b-3p overexpression inhibits the inflammatory STAT3 and NF-κB pathways in epidermal keratinocytes (Figs. 3 and S4). Unexpectedly, agomiR-193b-3p not only reduced the inflammatory response in epidermal keratinocytes but also inhibited the systemic immune response as indicated by decreased spleen size and splenic *Il-1β* expression (Fig. S4d, e, f). It is widely known that activated keratinocytes produce inflammatory mediators, thereby leading to the recruitment and activation of immune cells and consequently resulting in the expansion of psoriasis plaques [1–4]. Herein, disruption of the positive feedback loop between epidermal keratinocytes and immune cells via miR-193b-3p overexpression results in reduced epidermal hyperplasia and limited immune reaction, improving the systemic inflammation and immune response in the psoriasis-like mouse model.

As predicted via online tools and validated using a dual-luciferase reporter assay, *ERBB4* was demonstrated to be a direct target of miR-193b-3p (Figs. 5A,B and S6). Moreover, miR-193b-3p dramatically decreased the protein expression of ERBB4 in keratinocytes (Fig. 5C). However, neither miR-193b-3p mimics nor its inhibitors affected the mRNA levels of *ERBB4* in keratinocytes (Fig. 5D, E), indicating that miR-193b-3p regulates ERBB4 expression through a post-transcriptional mechanism.

The *ERBB4* gene is a member of the ERBB family and plays an important role in the occurrence and development of various tumors [28, 29]. Accumulating evidence has shown that ERBB4 plays a critical role in regulating cellular growth, as ERBB4 overexpression promotes proliferation and cellular survival, whereas ERBB4-null cells demonstrate decreased proliferation and cellular survival [40]. Study of its mechanism shows that ERBB4 promotes cell growth by activating signaling cascades including the STAT3, STAT5, and phospholipase-Cγ pathways [40]. Recently, Zhang et al. have reported that miR-302b inhibits cancer-related inflammation by regulating ERBB4, IRF2, and CXCR4 post-transcriptionally, while downregulation of these genes leads to decreased cytokine production, suggesting that ERBB4 plays a role in inflammation [41]. Meanwhile, ERBB4 is highly expressed in colorectal cancer cells and augments colorectal carcinogenesis [28]. Interestingly, in nontransformed colonocytes, inflammatory cytokines (e.g., TNF-α) could induce strong ERBB4 expression through a mechanism involving NF-κB signaling [42], while both TNF-α and NF-κB play crucial roles in psoriasis pathogenesis [34, 43–45]. In the skin epidermis, ERBB4 is highly expressed and affects the epidermal thickness [30]. In human keratinocyte cell lines, we had preliminary data showing that ERBB4 protein levels were increased by the M5 cocktail (Fig. S9). In the IMQ-induced mouse model and lesional skin samples from psoriasis patients, ERBB4 protein levels were significantly upregulated (Figs. 5G, H and S6d). However, the function of ERBB4 in psoriasis pathogenesis is not systematically studied. Herein, we used siRNAs/overexpression vector to examine its role in keratinocytes. As shown in Fig. 6, ERBB4 knockdown in keratinocytes simultaneously inhibited cellular proliferation and inflammatory gene expression, as well as the inflammatory STAT3 and NF-κB pathways. On the contrary, ERBB4 overexpression promoted proliferation and inflammatory-factor secretion in keratinocytes (Fig. 7). Notably, there were positive correlations between Erbb4/ERBB4 expression and PASI severity scores in the IMQ-induced mouse model and clinical samples (Fig. S6f). From the results above, we speculate that ERBB4 could promote the keratinocyte proliferation and inflammatory response through the STAT3 and NF-κB pathways, which might represent a novel role of ERBB4 in psoriasis pathogenesis. However, the precise mechanism of ERBB4 regulating psoriasis pathogenesis needs more in vivo data and clinical evidence to elucidate.

A gene recovery assay was performed to confirm the regulatory role of ERBB4. As shown in Fig. 8, ERBB4 overexpression completely abolished miR-193b-3p-mediated downregulation of cell proliferation and inflammatory-factor production in keratinocytes, further confirming that miR-193b-3p regulates the keratinocyte activities at least partially through targeting ERBB4.

**Fig. 9:**
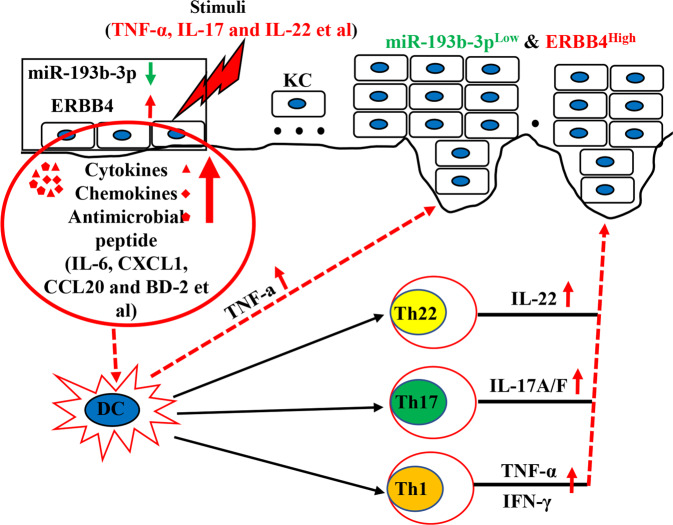
Schematic diagram of MiR-193b-3p/ERBB4 axis involved in the pathogenesis of psoriasis. When keratinocytes are in a psoriasis-related inflammatory environment (or are subjected to external stimuli), the expression of miR-193b-3p is downregulated, while that of ERBB4 is upregulated. ERBB4 promotes the proliferation and expression of inflammatory genes (*IL-6*, *CXCL1*, *CCL20*, *BD-2*, etc.) in keratinocytes, leading to the activation of keratinocytes and resulting in the recruitment and activation of relevant immune cells (including Th1, Th17, Th22, etc.). The activated immune cells secrete pro-inflammatory mediators (TNF-α, IL-17, IL-22, etc.), which further cause the downregulation of miR-193b-3p (ERBB4 is upregulated) in keratinocytes, forming a feedback loop and leading to the expansion of the inflammatory response in psoriasis.

In conclusion, our results demonstrate that miR-193b-3p acts as a negative regulator of psoriasis pathogenesis by directly targeting ERBB4. More importantly, functional inhibition of miR-193b-3p accelerated psoriasis development (Fig. 9), suggesting the potential use of therapeutic approaches targeting miR-193b-3p in psoriasis treatment. However, further in vivo experiments and studies are needed to confirm the clinical value of miR-193b-3p and ERBB4 in psoriasis.

## Supplementary information

Supplementary materials.

## Data Availability

The data underlying this article will be shared on reasonable request to the corresponding author.
