# Correction: MiR-193b-3p–ERBB4 axis regulates psoriasis pathogenesis via modulating cellular proliferation and inflammatory-mediator production of keratinocytes

**DOI:** 10.1038/s41419-021-04354-8

**Published:** 2021-11-10

**Authors:** Cong Huang, Weilong Zhong, Xuanyao Ren, Xia Huang, Zizhuo Li, Chaofeng Chen, Bin Jiang, Zhenzhen Chen, Xingling Jian, Lili Yang, Xiaoming Liu, Haiyan Huang, Changbing Shen, Xiaofan Chen, Xia Dou, Bo Yu

**Affiliations:** 1 Department of Dermatology, Skin Research Institute of Peking University Shenzhen Hospital, Peking University Shenzhen Hospital, Shenzhen Peking University - The Hong Kong University of Science and Technology Medical Center, Shenzhen, 518036 China; 2 Department of Dermatology, Skin Research Institute of Peking University Shenzhen Hospital, Peking University Shenzhen Hospital, Shenzhen, 518036 China; 3 Biomedical Research Institute, Shenzhen Peking University - The Hong Kong University of Science and Technology Medical Center, Shenzhen, 518036 China

**Keywords:** Mechanisms of disease, miRNAs, Mechanisms of disease, miRNAs

Correction to: *Cell Death and Disease* (2021) 10.1038/s41419-021-04230-5, published online 19 October 2021

The original version of this article unfortunately contained a mistake in the author affiliations. Due to a typesetting error the affiliations for the first author and the corresponding author were changed after manuscript proof. We apologize for the mistake. The original article has been corrected.

