# Supplementary material for: MiR-193b-3p–ERBB4 axis regulates psoriasis pathogenesis via modulating cellular proliferation and inflammatory-mediator production of keratinocytes
Source: Cell Death Dis. 2021 Oct 19;12(11):963. doi: 10.1038/s41419-021-04230-5 (PMC8526743; doi:10.1038/s41419-021-04230-5)
Supplement: Supplementary file 1 — Supplementary materials. [file 41419_2021_4230_MOESM1_ESM.pdf]

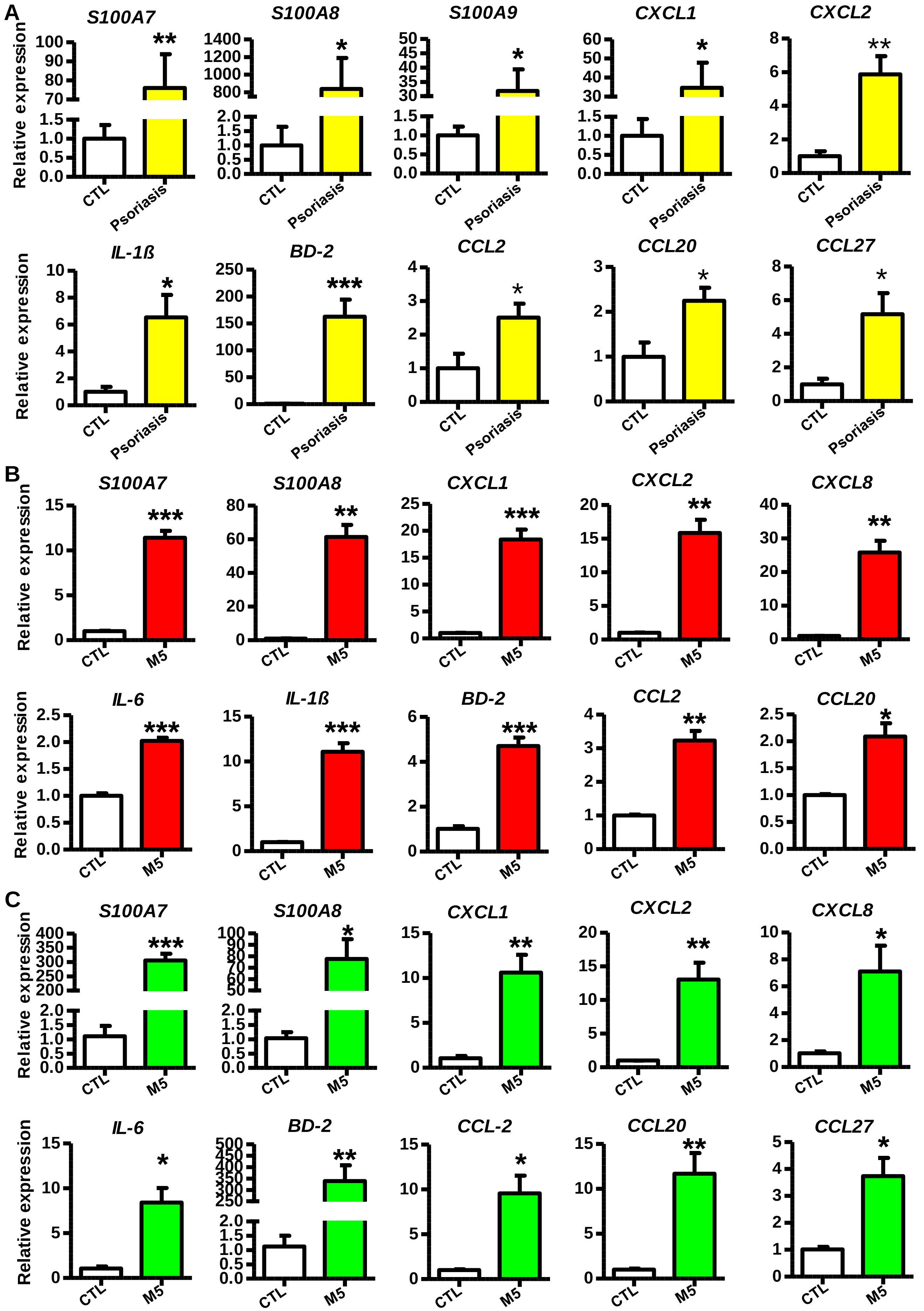
A
S100A7
S100A8
S100A9
CXCL1
CXCL2
IL-1ß
BD-2
CCL2
CCL20
CCL27
Relative expression
CTL
Psoriasis
B
S100A7
S100A8
CXCL1
CXCL2
CXCL8
IL-6
IL-1ß
BD-2
CCL2
CCL20
Relative expression
CTL
M5
C
S100A7
S100A8
CXCL1
CXCL2
CXCL8
IL-6
BD-2
CCL-2
CCL20
CCL27
Relative expression
CTL
M5

Fig S1

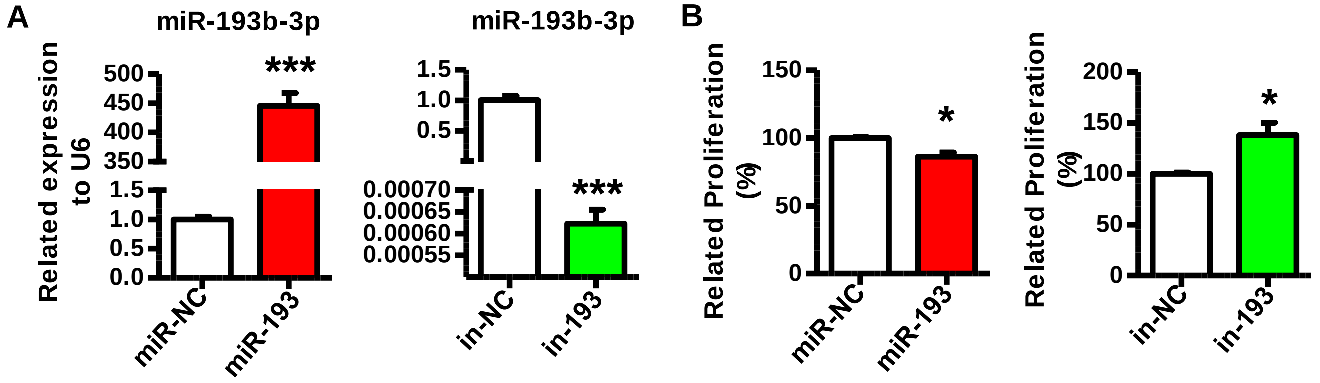
A
B
miR-193b-3p
miR-193b-3p
Related expression to U6
500
450
400
350
1.5
1.0
0.5
0.0
***
miR-NC
miR-193
1.5
1.0
0.5
0.00070
0.00065
0.00060
0.00055
***
in-NC
in-193
Related Proliferation (%)
150
100
50
0
*
miR-NC
miR-193
Related Proliferation (%)
200
150
100
50
0
*
in-NC
in-193

Fig S2

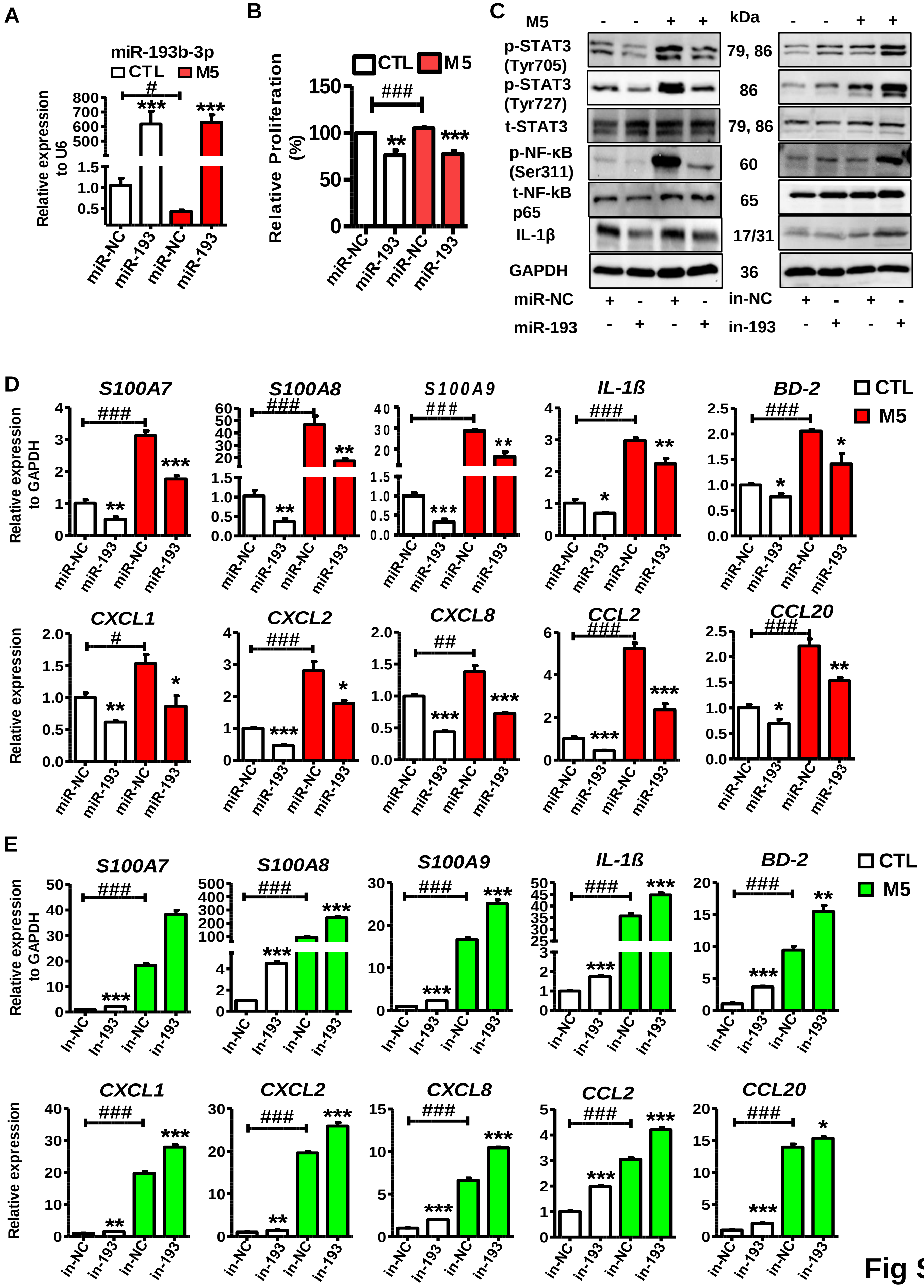
A
miR-193b-3p
CTL
M5
Relative expression to U6
miR-NC
miR-193
B
Relative Proliferation (%)
C
M5
kDa
p-STAT3 (Tyr705)
p-STAT3 (Tyr727)
t-STAT3
p-NF-κB (Ser311)
t-NF-kB p65
IL-1β
GAPDH
79, 86
86
79, 86
60
65
17/31
36
miR-NC
miR-193
in-NC
in-193
D
S100A7
S100A8
S100A9
IL-1ß
BD-2
CXCL1
CXCL2
CXCL8
CCL2
CCL20
Relative expression to GAPDH
Relative expression
E
in-NC
in-193

Fig S3

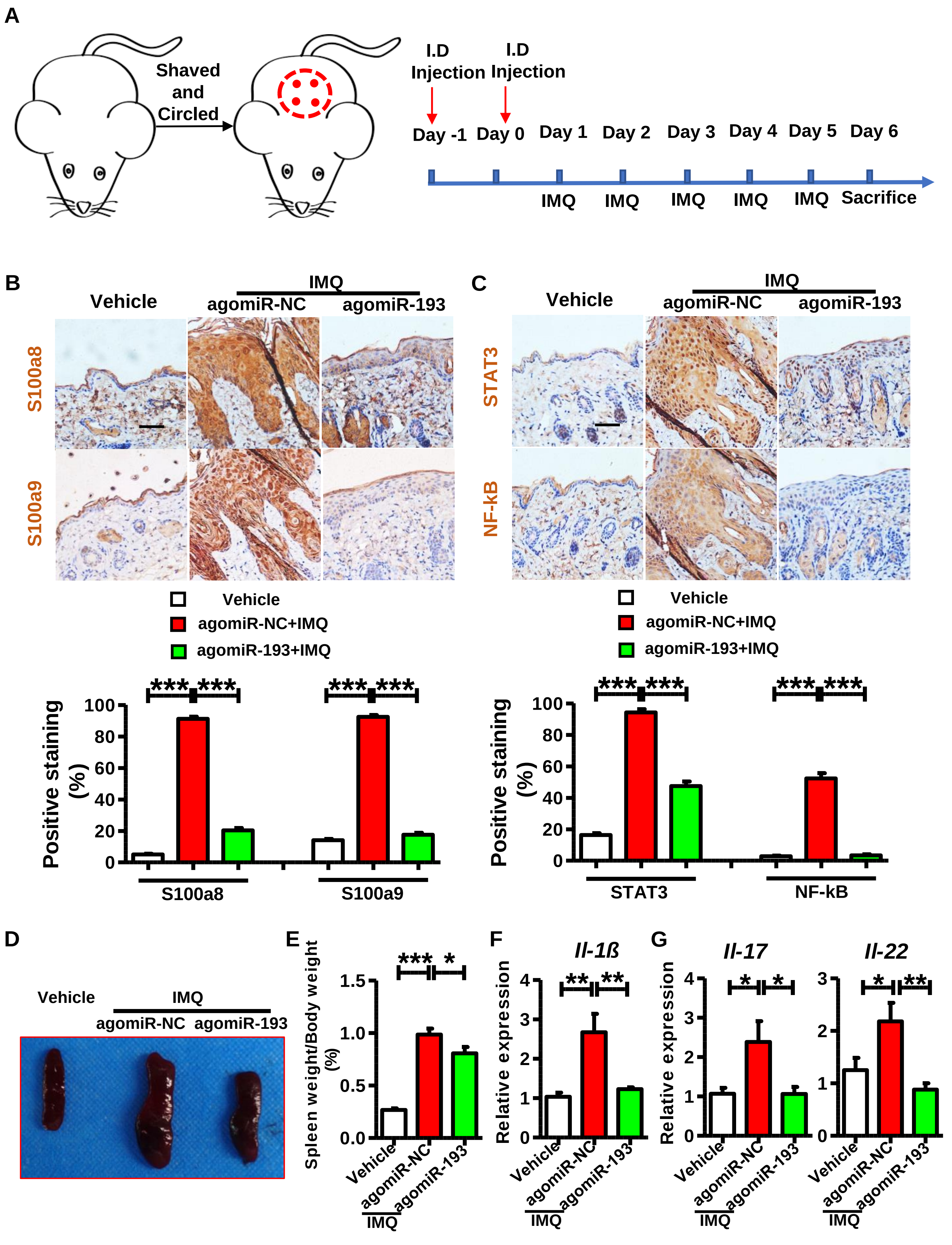
A
Shaved and Circled
I.D Injection
I.D Injection
Day -1
Day 0
Day 1
Day 2
Day 3
Day 4
Day 5
Day 6
IMQ
IMQ
IMQ
IMQ
IMQ
Sacrifice
B
Vehicle
IMQ
agomiR-NC
agomiR-193
S100a8
S100a9
C
STAT3
NF-kB
Vehicle
agomiR-NC+IMQ
agomiR-193+IMQ
Positive staining (%)
100
80
60
40
20
0
S100a8
S100a9
STAT3
NF-kB
D
E
Spleen weight/Body weight (%)
1.5
1.0
0.5
0.0
F
Il-1ß
Relative expression
G
Il-17
Il-22
Vehicle
agomiR-NC
agomiR-193
IMQ

Fig S4

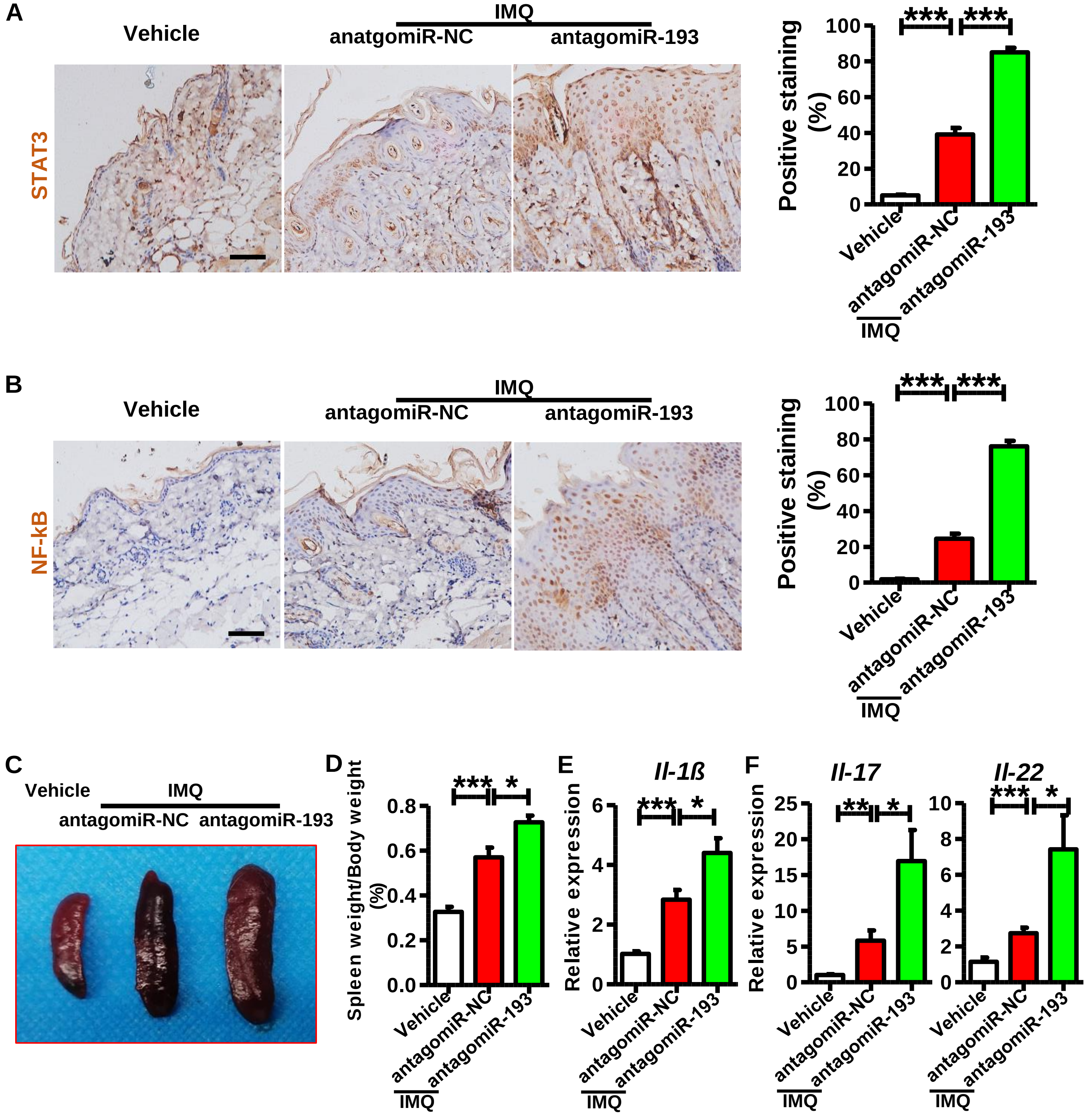
A
Vehicle
IMQ
anatgomiR-NC
antagomiR-193
STAT3
Positive staining (%)
***
***
Vehicle
antagomiR-NC
antagomiR-193
IMQ
B
Vehicle
IMQ
antagomiR-NC
antagomiR-193
NF-kB
Positive staining (%)
C
Vehicle
IMQ
antagomiR-NC antagomiR-193
D
Spleen weight/Body weight (%)
*
E
Il-1ß
Relative expression
F
Il-17
Il-22
**

Fig S5

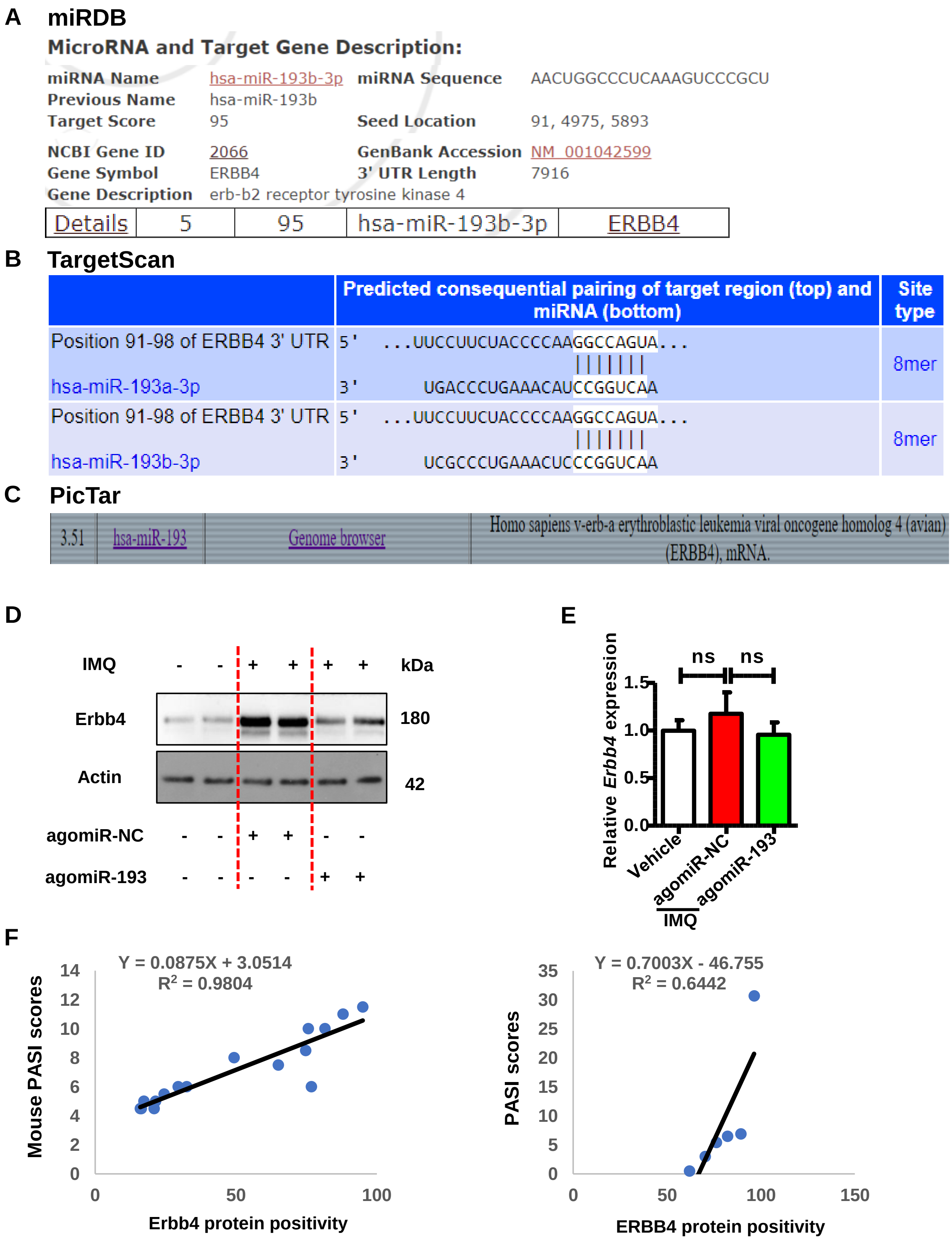
A
miRDB
MicroRNA and Target Gene Description:
miRNA Name hsa-miR-193b-3p
miRNA Sequence AACUGGCCCUCAAAGUCCCGCU
Previous Name hsa-miR-193b
Target Score 95
Seed Location 91, 4975, 5893
NCBI Gene ID 2066
GenBank Accession NM_001042599
Gene Symbol ERBB4
3' UTR Length 7916
Gene Description erb-b2 receptor tyrosine kinase 4
Details 5 95 hsa-miR-193b-3p ERBB4
B
TargetScan
Predicted consequential pairing of target region (top) and miRNA (bottom)
Site type
Position 91-98 of ERBB4 3' UTR
hsa-miR-193a-3p
5' ...UUCCUUCUACCCCAAGGCCAGUA...
3' UGACCCUGAAACAUCCGGUCAA
8mer
Position 91-98 of ERBB4 3' UTR
hsa-miR-193b-3p
5' ...UUCCUUCUACCCCAAGGCCAGUA...
3' UCGCCCUGAAACUCCCGGUCAA
8mer
C
PicTar
3.51 hsa-miR-193 Genome browser Homo sapiens v-erb-a erythroblastic leukemia viral oncogene homolog 4 (avian) (ERBB4), mRNA.
D
IMQ - - + + + +
kDa
Erbb4 180
Actin 42
agomiR-NC - - + + - -
agomiR-193 - - - - + +
E
Relative Erbb4 expression
ns ns
0.0 0.5 1.0 1.5
Vehicle
agomiR-NC
agomiR-193
IMQ
F
Y = 0.0875X + 3.0514
R² = 0.9804
Mouse PASI scores
0 2 4 6 8 10 12 14
0 50 100
Erbb4 protein positivity
Y = 0.7003X - 46.755
R² = 0.6442
PASI scores
0 5 10 15 20 25 30 35
0 50 100 150
ERBB4 protein positivity

Fig S6

A

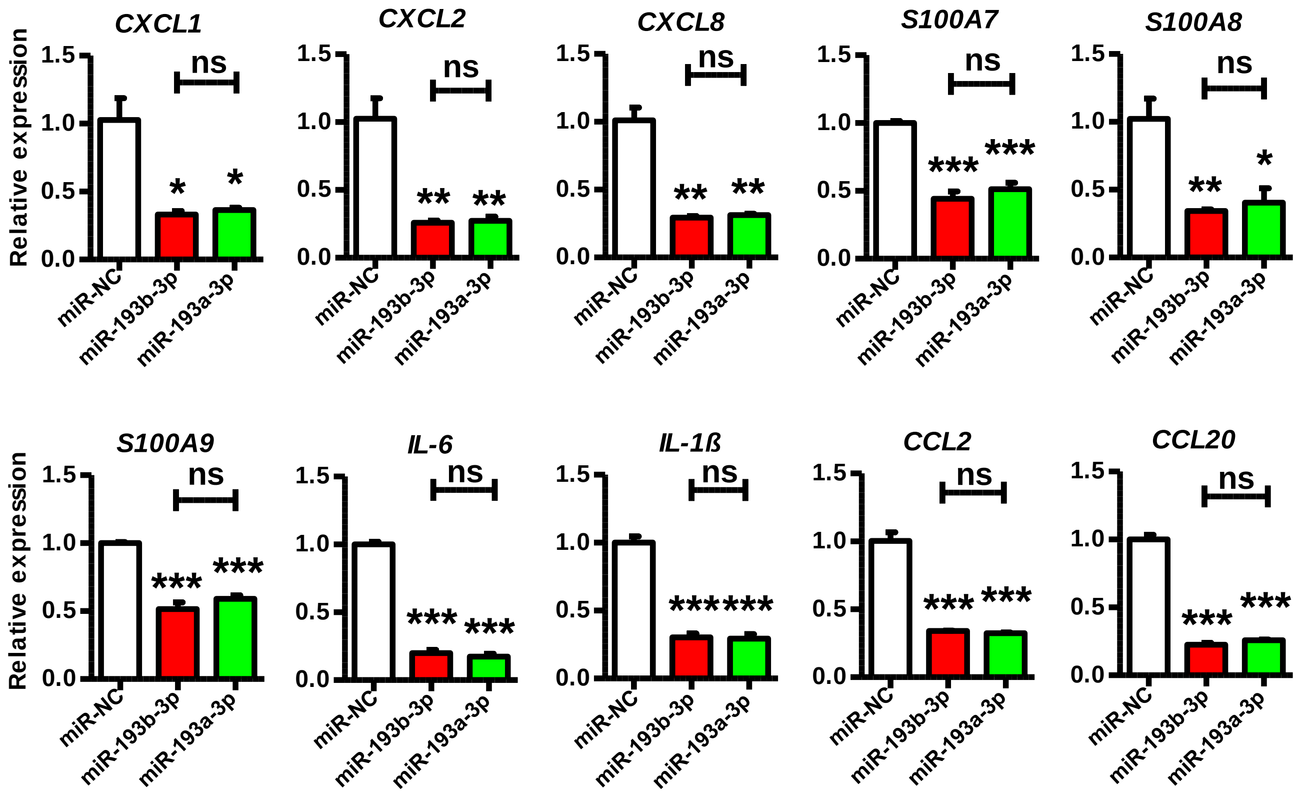

CXCL1
CXCL2
CXCL8
S100A7
S100A8
S100A9
IL-6
IL-1ß
CCL2
CCL20
Relative expression
ns
miR-NC
miR-193b-3p
miR-193a-3p

Fig S7

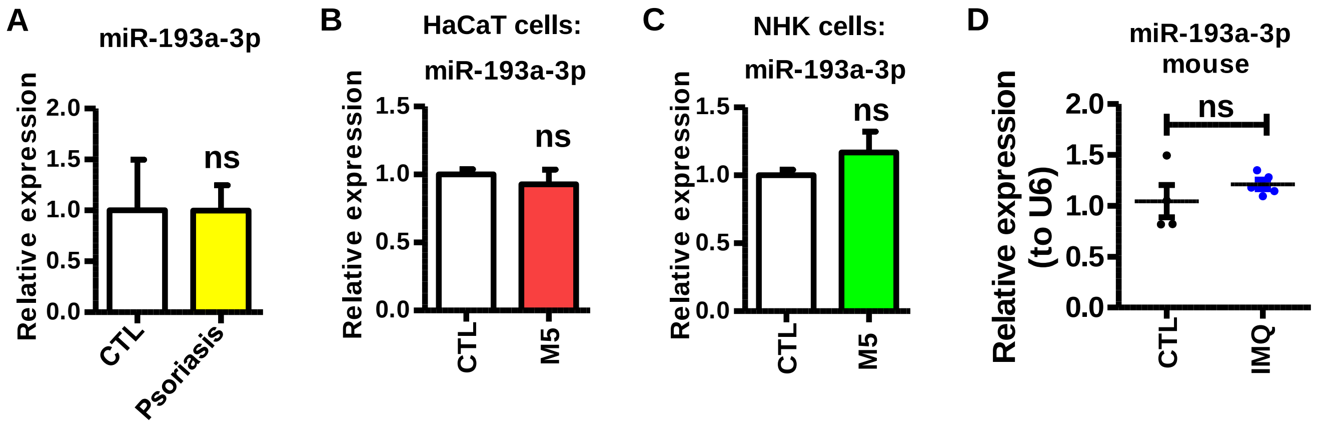
A
miR-193a-3p
Relative expression
2.0
1.5
1.0
0.5
0.0
ns
CTL
Psoriasis
B
HaCaT cells:
miR-193a-3p
Relative expression
1.5
1.0
0.5
0.0
ns
CTL
M5
C
NHK cells:
miR-193a-3p
Relative expression
1.5
1.0
0.5
0.0
ns
CTL
M5
D
miR-193a-3p
mouse
Relative expression
(to U6)
2.0
1.5
1.0
0.5
0.0
ns
CTL
IMQ

Fig S8

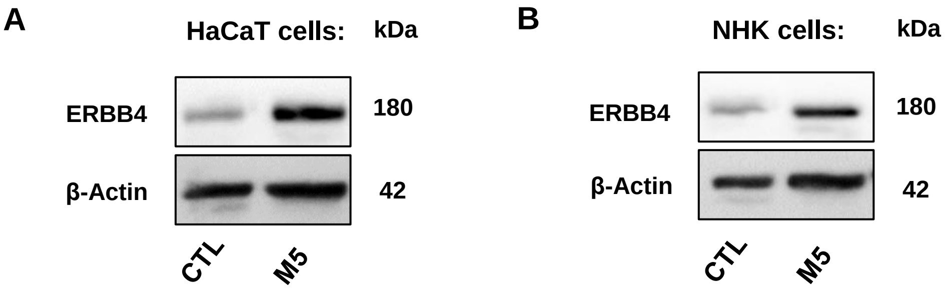
A
HaCaT cells:
kDa
ERBB4
180
β-Actin
42
CTL
M5
B
NHK cells:
kDa
ERBB4
180
β-Actin
42
CTL
M5

Fig S9

Supplementary Figure Legends

Figure S1. Expression of psoriasis-related genes and inflammatory genes in psoriasis patients and M5-induced keratinocytes.

A Expression of psoriasis-related genes and inflammatory genes in skin tissues derived from health donors (CTL, n=5) and psoriasis patients (Psoriasis, n=6). mRNA levels were analyzed using qRT-PCR. Each bar represents the Mean ± SEM. ***: $P < 0.001$, **: $P < 0.01$, *: $P < 0.05$, compared with the indicated controls.

B Expression of psoriasis-related genes and inflammatory genes in HaCat cells with or without M5-treatment. mRNA levels were analyzed using qRT-PCR. Each bar represents the Mean ± SEM (n=3). ***: $P < 0.001$, **: $P < 0.01$, *: $P < 0.05$, compared with the indicated controls.

C Expression of psoriasis-related genes and inflammatory genes in normal human keratinocytes (NHKs) with or without M5-treatment. mRNA levels were analyzed using qRT-PCR. Each bar represents the Mean ± SEM (n=3). ***: $P < 0.001$, **: $P < 0.01$, *: $P < 0.05$, compared with the indicated controls.

Figure S2. Role of miR-193b-3p in HaCaT cells.

A Expression of miR-193b-3p in HaCaT cells transfected with miR-193b-3p mimics (miR-193) or miR-193b-3p inhibitors (in-193) and their indicated CTLs (miR-NC and in-NC). Cells were collected 2 days after transfection and mRNA levels were analyzed using qRT-PCR. U6 was used as the internal control. Each bar represents the Mean ± SEM (n=3). ***: $P < 0.001$, compared with the indicated controls.

B Relative proliferation rate of HaCaT cells transfected with miR-193b-3p mimics (miR-193) or miR-193b-3p inhibitors (in-193) and their indicated CTLs (miR-NC and in-NC). Cell proliferation was analyzed using MTT assay. Each bar represents the Mean ± SEM (n=3). *: $P < 0.05$, compared with the indicated controls.

Figure S3. Role of miR-193b-3p in M5-induced normal human keratinocytes (NHKs).

A Expression of miR-193b-3p in NHKs transfected with miR-193b-3p mimics (miR-193)/indicated CTL (miR-NC) in the presence or absence of M5 treatment. Cells were collected after 2 days' transfection plus 24 hours' M5/PBS induction. mRNA levels were analyzed using qRT-PCR. U6 was used as the internal control. Each bar represents the Mean ± SEM (n=4). ***: $P < 0.001$, #: $p < 0.05$, compared with the indicated controls.

B Relative proliferation rate of NHKs transfected with miR-193b-3p mimics (miR-193)/CTL (miR-NC) in the presence or absence of M5 treatment. Cell proliferation was analyzed using MTT assay. Each bar represents the Mean ± SEM (n=4). ***: $P < 0.001$, **: $P < 0.01$, compared with the indicated controls.

C Representative blots showing protein levels of phospho-STAT3 (Tyr705), phospho-STAT3 (Ser727), phospho-NF-kB p65 (Ser311), and IL-1β et al. in NHKs transfected with miR-193b-3p mimics (miR-193) or miR-193b-3p inhibitors (in-193) and their indicated CTLs (miR-NC/in-NC) in the presence or absence of M5 treatment. Protein levels were detected by western blotting. GAPDH was used as a protein loading control.

D Expression of psoriasis-related genes and inflammatory genes in NHKs transfected with miR-193b-3p mimics (miR-193)/miR-NC in the presence or absence of M5 treatment. Cells were collected after 2 days' transfection plus 24 hours' M5/PBS treatment. mRNA levels were analyzed using qRT-PCR. GAPDH was used as the internal control. Each bar represents the Mean ± SEM (n=4). ***: $P < 0.001$, **: $P < 0.01$, *: $P < 0.05$, ###: $p < 0.001$, ##: $p < 0.01$, #: $p < 0.05$, compared with the indicated controls.

E Expression of psoriasis-related genes and inflammatory genes in NHKs transfected with miR-193b-3p inhibitors (in-193)/in-NC in the presence or absence of M5 treatment. Cells were collected after 2 days' transfection plus 24 hours' M5/PBS treatment. mRNA levels were analyzed using qRT-PCR. GAPDH was used as the internal control. Each bar represents the Mean ± SEM (n=4). ***: $P < 0.001$, **: $P < 0.01$, *: $P < 0.05$, ###: $p < 0.001$, compared with the indicated controls.

Figure S4. Role of agomiR-193b-3p in IMQ-induced psoriasis mouse model.

A Schematic experimental protocol showing treatment regimens using agomiR-193b-3p in IMQ-induced psoriasis mice (n=8 for each group).

B Upper panel: Representative images showing expression of S100a8 and S100a9 in the skin tissues derived from mouse back injected with agomiR-193b-3p (agomiR-193) or indicated CTL (agomiR-NC) treated with IMQ or vehicle for 5 days. Scar bar: 100 μm. Lower panel: Quantification of S100a8 and S100a9 positivity in the skin tissues derived from different treatment groups. Each bar represents the Mean ± SEM (n=8 for each group). ***: $P < 0.001$, compared with the indicated controls.

C Upper panel: Representative images showing expression of STAT3 and NF-kB in the skin tissues derived from mouse back injected with agomiR-193/agomiR-NC treated with IMQ or vehicle for 5 days. Scar bar: 100 μm. Lower panel: Quantification of STAT3 and NF-kB positivity in the skin tissues derived from different treatment groups. Each bar represents the Mean ± SEM (n=8 for each group). ***: $P < 0.001$, compared with the indicated controls.

D Representative pictures showing the spleens-derived from mice injected with agomiR-193 or agomiR-NC treated with IMQ or vehicle for 5 days.

E Quantification data of spleen weight/mouse body weight in different groups. Each bar represents the Mean ± SEM (n=8 for each group). ***: $P < 0.001$, *: $P < 0.05$ compared with the indicated controls.

F mRNA levels of *Il-1β* in spleens derived from mice injected with agomiR-193 or agomiR-NC in the presence or absence of IMQ treatment for 5 consecutive days. Spleen tissues were collected at day 6 and mRNA levels were analyzed using qRT-PCR. Gapdh was used as the internal control. Each bar represents the Mean ± SEM (n=8 for each group). **: P < 0.01 compared with the indicated controls.

G mRNA levels of *Il-17* and *Il-22* in lymph nodes derived from mice injected with agomiR-193 or agomiR-NC in the presence or absence of IMQ treatment for 5 consecutive days. Lymph nodes were collected at day 6 and mRNA levels were analyzed using qRT-PCR. Gapdh was used as the internal control. Each bar represents the Mean ± SEM (n=8 for each group). **: P < 0.01, *: P < 0.05 compared with the indicated controls.

Figure S5. Role of antagomiR-193b-3p in IMQ-induced psoriasis mouse model.

A Left panel: Representative images showing expression of STAT3 in the skin tissues derived from mouse back injected with antagomiR-193b-3p/antagomiR-NC treated with IMQ or vehicle for 5 days. Scar bar: 100 μm. Right panel: Quantification of STAT3 positivity in the skin tissues derived from different groups. Each bar represents the Mean ± SEM (n=8 for each group). ***: P < 0.001, compared with the indicated controls.

B Left panel: Representative images showing expression of NF-kB in the skin tissues derived from mouse back injected with antagomiR-193b-3p/antagomiR-NC treated with IMQ or vehicle for 5 days. Scar bar: 100 μm. Right panel: Quantification of NF-kB positivity in the skin tissues derived from different groups. Each bar represents the Mean ± SEM (n=8 for each group). ***: P < 0.001, compared with the indicated controls.

C Representative pictures showing the spleens-derived from mice injected with antagomiR-193b-3p or antagomiR-NC treated with IMQ or vehicle for 5 days.

D Quantification data of spleen weight/mouse body weight in different groups. Each bar represents the Mean ± SEM (n=8 for each group). ***: P < 0.001, *: P < 0.05 compared with the indicated controls.

E mRNA levels of *Il-1β* in spleens derived from mice injected with antagomiR-193b-3p or antagomiR-NC in the presence or absence of 5 consecutive days' IMQ treatment. Spleen tissues were collected at day 6 and mRNA levels were analyzed using qRT-PCR. Gapdh was used as the internal control. Each bar represents the Mean ± SEM (n=8 for each group). **: P < 0.01 compared with the indicated controls.

F mRNA levels of *Il-17* and *Il-22* in lymph nodes derived from mice injected with antagomiR-193b-3p or antagomiR-NC in the presence or absence of IMQ treatment. Lymph nodes were collected at day 6 and mRNA levels were analyzed using qRT-PCR. Gapdh was used as the internal control. Each bar represents the Mean ± SEM (n=8 for each group). **: P < 0.01, *: P < 0.05 compared with the indicated controls.

Figure S6. miR-193b-3p is predicted to target the 3' UTR of ERBB4 and modulates its expression post-transcriptionally.

A The 3' UTR of ERBB4 mRNA was predicted to be targeted by miR-193b-3p using miRDB website.

B ERBB4 was predicted to be a potential target gene of miR-193b-3p using TargetScan.

C ERBB4 was one of the candidate target genes of miR-193b-3p predicted by PicTar.

D Representative blots showing protein levels of Erbb4 in skin tissues derived from mice injected with agomiR-193 or agomiR-NC in the presence or absence of IMQ treatment for 5 consecutive days. Protein levels were detected by western blotting. GAPDH was used as a protein loading control.

E mRNA levels of *Erbb4* in skin tissues derived from mice injected with agomiR-193 or agomiR-NC in the presence or absence of IMQ treatment for 5 consecutive days. mRNA levels were analyzed using qRT-PCR. Gapdh was used as the internal control. Each bar represents the Mean ± SEM (n=8 for each group). ns: no significant difference compared with the indicated controls.

F Left panel: Correlation of mouse PASI scores and Erbb4 protein positivity in IMQ-induced psoriasis mouse model (n=16). Right panel: Correlation of patient PASI scores and ERBB4 protein positivity in clinic samples (n=6).

Figure S7. miR-193b-3p and miR-193a-3p share similar function regarding the regulation of inflammatory-factor production in keratinocytes.

A mRNA levels of psoriasis-related genes and inflammatory genes in keratinocytes transfected with miR-193b-3p mimics (miR-193b-3p) or miR-193a-3p mimics (miR-193a-3p) or indicated CTL (miR-NC). Cells were collected 2 days after transfection and mRNA levels were analyzed using qRT-PCR. GAPDH was used as the internal control. Each bar represents the Mean ± SEM (n=3). ***: $P < 0.001$, **: $P < 0.01$, *: $P < 0.05$, ns: no significant difference compared with the indicated controls.

Figure S8. Expression profiles of miR-193a-3p in psoriasis patients, M5-induced keratinocytes, and IMQ-induced mouse model.

A Expression levels of miR-193a-3p in skin tissues derived from health donor (CTL, n=5) and psoriasis patients (Psoriasis, n=6). mRNA levels were analyzed using qRT-PCR. U6 was used as the internal control. Each bar represents the Mean ± SEM. ns: no significant difference, compared with the indicated controls.

B Expression levels of miR-193a-3p in HaCat cells in the presence or absence of M5-treatment. mRNA levels were analyzed using qRT-PCR. U6 was used as the internal control. Each bar represents the Mean ± SEM (n=3). ns: no significant difference, compared with the indicated controls.

C Expression levels of miR-193a-3p in normal human keratinocytes (NHKs) in the presence or absence of M5-treatment. mRNA levels were analyzed using qRT-PCR. U6 was used as the internal control. Each bar represents the Mean ± SEM (n=3). ns: no significant difference, compared with the indicated controls.
D Expression levels of miR-193a-3p in the back skin of CTL (Vaseline) and IMQ-treated mice. mRNA levels were analyzed using qRT-PCR. U6 was used as the internal control. Each bar represents the Mean ± SEM (n≥4). ns: no significant difference, compared with the indicated controls.

Figure S9. Protein levels of ERBB4 in M5-induced keratinocytes.
A Representative blots showing protein levels of ERBB4 in HaCat cells in the presence (M5) or absence (CTL) of M5-treatment. Protein levels were detected by western blotting. β-Actin was used as a protein loading control.
B Representative blots showing protein levels of ERBB4 in normal human keratinocytes (NHKs) in the presence (M5) or absence (CTL) of M5-treatment. Protein levels were detected by western blotting. β-Actin was used as a protein loading control.

Table S1 Primer sequences used for qRT-PCR in this study

Gene names	Sequences (5'-3')
Human genes	
hS100A7-Fw	CCTTAGTGCCTGTGACAA
hS100A7-Rev	CTGCTTGTGGTAGTCTGT
hS100A8-Fw	AGTGTCCTCAGTATATCA
hS100A8-Rev	CATCTTTATCACCAGAATG
hS100A9-Fw	CAACACCTTCCACCAATAC
hS100A9-Rev	TCATTCTTATTCTCCTTCTTGAG
hCXCL1-Fw	GCCAGTGCTTGCAGACCCT
hCXCL1-Rev	GGCTATGACTTCGGTTTGGG
hCXCL2-Fw	CAAACCGAAGTCATAGCCAC
hCXCL2-Rev	TCTGGTCAGTTGGATTTGCC
hCXCL8-Fw	TCTGTCTGGACCCCAAGGAA
hCXCL8-Rev	GCATCTGGCAACCCTACAACA
hBD2-Fw	TTCTCGTTCCTCTTCATA
hBD2-Rev	ATATGGCTCCACTCTTAA
hIL-6-Fw	GGCACTGGCAGAAAACAACC
hIL-6-Rev	GCAAGTCTCCTCATTGAATCC
hCCL2-Fw	CTTCTGTGCCTGCTGCTC
hCCL2-Rev	TGCTGCTGGTGATTCTTCT
hCCL20-Fw	TGACTGCTGTCTTGGATACACAGA
hCCL20-Rev	TGATAGCATTGATGTCACAGCCT
hCCL27-Fw	AGCACTGCCTGCTGTACTCA
hCCL27-Rev	TCTTGGTGCTCAAACCACTG
hIL-1b-Fw	AGCTACGAATCTCCGACCAC
hIL-1b-Rev	CGTTATCCCATGTGTCGAAGAA
hERBB4-Fw	GAGCAAGAATTGACTCGAATAGG
hERBB4-Rev	TTCCTGACATGGGGGTGTAG
hGAPDH-Fw	GGTGTGAACCATGAGAAGTATGA
hGAPDH-Rev	GAGTCCTTCCACGATACCAAAG
Mouse genes	
mS100a7-Fw	ACATCACGGACTGGCAGAACGT
mS100a7-Rev	AGGATGTCGTGGAACTGGTCAG
mS100a8-Fw	TCCTTGCGATGGTGATAAA
mS100a8-Rev	GGCCAGAAGCTCTGCTACTC
mS100a9-Fw	GACACCCTGACACCCTGAG
mS100a9-Rev	TGAGGGCTTCATTTCTCTTCTC
mCxcl1-Fw	CACAGGGGCGCCTATCGCCAA
mCxcl1-Rev	CAAGGCAAGCCTCGCGACCAT
mCxcl8-Fw	CAAGGCTGGTCCATGCTCC
mCxcl8-Rev	TGCTATCACTTCCTTTCTGTTGC
mCcl20-Fw	GCCTCTCGTACATACAGACGC
mCcl20-Rev	CCAGTTCTGCTTTGGATCAGC
mIl-1b-Fw	GCACTACAGGCTCCGAGATGAAC
mIl-1b-Rev	TTGTCGTTGCTTGGTTCTCCTTGT
mIl-17a-Fw	CCAGGGAGAGCTTCATCTGT
mIl-17a-Rev	ACGTGGAACGGTTGAGGTAG
mIl-17f-Fw	TTGATGCAGCCTGAGTGTCT
mIl-17f-Rev	AATTCCAGAACCGCTCCAGT

mIl-22-Fw	GTGACGACCAGAACATCCAGAA
mIl-22-Rev	CCCCAATCGCCTTGATCTCT
mIfng-Fw	AGGAACTGGCAAAAGGATGGT
mIfng-Rev	TCATTGAATGCTTGGCGCTG
mTnfa-Fw	CTGTAGCCCACGTCGTAGC
mTnfa-Rev	TTAAGATCCATGCCGTTG
mErbb4-Fw	TTGCCATCCAAACTGCACC
mErbb4-Rev	TCCAATGACTCCGGCTGC
mGapdh-Fw	CATCACTGCCACCCAGAAGACTG
mGapdh-Rev	ATGCCAGTGAGCTTCCCGTTCAG

Table S2 Details of miR-193b-3p/miR-193a-3p gene locations and mature sequences in *Homo sapiens* and *Mus musculus*

miRNA IDs	Accession number	Chromosome	Sequences
Has-miR-193b-3p	MIMAT0002819	Chr16	AACUGGCCCUCAAAGUCCCGCU
Mmu-miR-193b-3p	MIMAT0004859	Chr16	AACUGGCCCACAAAGUCCCGCU
Has-miR-193a-3p	MIMAT0000459	Chr17	AACUGGCCUACAAAGUCCCAGU
Mmu-miR-193a-3p	MIMAT0000223	Chr11	AACUGGCCUACAAAGUCCCAGU
